# Polymer-Grafted WS_2_ Nanocomposites: from Edge-Site Passivation to Melt-Stable Fused Granular Fabrication

**DOI:** 10.1021/acsnano.5c22326

**Published:** 2026-04-21

**Authors:** Mirko Maturi, Alberto Sanz de Leon, Luisa M. Valencia, Sergio I. Molina, Miriam Herrera

**Affiliations:** † Dpto. Ciencia de los Materiales, I. M. y Q. I., IMEYMAT, Facultad de Ciencias, 16727 Universidad de Cádiz, Campus Río San Pedro, s/n, 11510 Puerto Real, Cádiz, Spain; ‡ Instituto de Ciencia de Materiales de Sevilla (CSIC-US), Americo Vespucio 49, Seville 41092, Spain

**Keywords:** edge-site catalysis, transition metal dichalcogenides, polymer degradation, surface-initiated polymer grafting, melt-processed nanocomposites

## Abstract

Layered transition metal dichalcogenides (TMDs) are widely regarded as chemically inert nanofillers in polymer composites. Here, we demonstrate that this assumption fails under melt-processing conditions. We show that pristine WS_2_ nanopowders act as heterogeneous catalysts for polyester chain scission during melt extrusion, inducing a catastrophic, 10-fold reduction in molecular weight, severe loss of melt viscosity, printing failure, and brittle mechanical behavior at filler loadings as low as 0.2 wt %. To suppress this unexpected catalytic activity, we develop an edge- and defect-selective functionalization strategy for WS_2_ based on covalent carboxylation and hydroxylation, followed by surface-initiated ring-opening polymerization of ε-caprolactone. Spectroscopic and microscopic analyses (XPS, XRD, HAADF-STEM, and EELS) demonstrate that polymer grafting is confined to edge and defect sites, while preserving the multilayer 2H-WS_2_ lattice. When incorporated into a polycaprolactone (PCL) matrix and processed by large-format fused granular fabrication, polymer-grafted WS_2_ nanostructures exhibit stable melt rheology, excellent printability, and substantial mechanical reinforcement, with Young’s modulus and tensile strength increases up to 45% and 65%, respectively, without loss of ductility. Crucially, polymer grafting effectively passivates catalytically active WS_2_ edge and defect sites, preventing melt-induced polymer degradation. These findings provide direct experimental evidence that exposed edge sites in layered nanomaterials can actively catalyze polymer degradation under melt-processing conditions and establish edge-site passivation as a general design principle to mitigate chemically driven polymer degradation in polyester-based systems during melt processing, with the magnitude of the effect depending on the chemical susceptibility of the host polymer.

## Graphical abstract

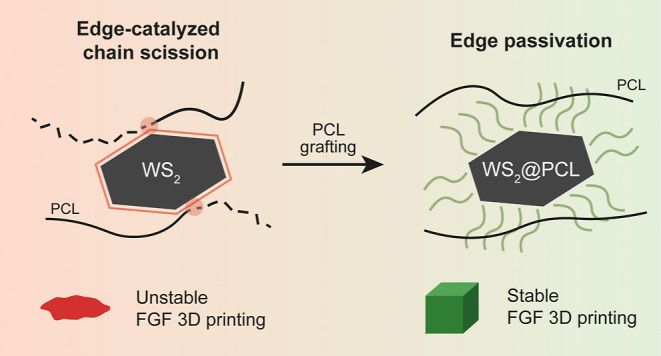

## Introduction

Layered transition metal dichalcogenides have emerged as a versatile class of nanomaterials for polymer-based composites, owing to their two-dimensional morphology, high intrinsic stiffness, thermal stability, and rich surface chemistry.^1,2^ Among them, tungsten disulfide (WS_2_) has received increasing attention due to its favorable balance between mechanical performance, perceived chemical robustness, and scalability of production.^3^ WS_2_ is commercially available in large quantities in the form of multilayered nanoplatelets and micropowders, making it particularly attractive for applications beyond laboratory-scale demonstrations, including structural and semistructural polymer components. In contrast to oxide fillers, whose surface acidity or redox activity is widely recognized in polymer processing, WS_2_ is commonly regarded as a chemically inert additive under melt-processing conditions, particularly when used in multilayer powder form.^4–6^

In polymer composites, WS_2_ has been investigated as a reinforcing filler, solid lubricant, and multifunctional additive, with reported improvements in stiffness, wear resistance and, in selected cases, thermal and electrical properties.^7–9^ These benefits are commonly attributed to the high aspect ratio of the layered filler and its ability to interact with the surrounding polymer matrix. However, most literature reports focus on solution-processed systems, low filler loadings, or qualitative assessments of dispersion, while comparatively little attention has been devoted to the behavior of WS_2_ under melt-processing conditions.^5,10^ This represents a significant knowledge gap, particularly in view of the growing importance of extrusion-based manufacturing technologies. This implicit assumption of chemical passivity is especially critical for layered materials such as WS_2_, whose apparent robustness is often extrapolated from basal-plane chemistry without considering edge- and defect-mediated reactivity.

Melt processing, including twin-screw extrusion and fused deposition-based additive manufacturing, subjects polymer–filler systems to elevated temperatures, high shear stresses, and extended residence times.^11–13^ Under these conditions, not only physical dispersion but also chemical compatibility and stability become critical. Even subtle interfacial reactions or catalytic effects can profoundly alter polymer molecular weight, melt viscosity, and ultimately processability and mechanical performance.^14,15^ The chemical role of inorganic nanofillers during melt processing is often implicitly assumed to be passive. However, this assumption is fundamentally flawed for layered materials whose edge and defect sites are intrinsically undercoordinated and chemically active.^16,17^

From a structural and chemical perspective, this assumption is questionable. TMDs exhibit a highly anisotropic reactivity: while the basal planes of WS_2_ are chemically saturated and largely inert, the edge and defect sites (including defect-associated regions such as vacancies and grain boundaries) are undercoordinated and chemically active.^18,19^ In multilayer WS_2_ nanoplatelets, such reactive sites are intrinsically present at sheet edges, step defects, and grain boundaries, and their relative abundance increases as particle size decreases.^20,21^ As a result, WS_2_ nanopowders cannot be regarded as uniformly inert fillers, particularly under melt-processing conditions where intimate contact with polymer chains is enforced. While other layered chalcogenides share a similar basal-plane/edge-site anisotropy, the density, chemical nature, and accessibility of reactive edge and defect sites can vary significantly across materials, making WS_2_ an ideal model system to isolate and study edge-mediated effects under melt-processing conditions.

At the same time, poor interfacial compatibility between WS_2_ and most thermoplastic polymers further complicates their integration. The lack of specific interactions often leads to aggregation, stress concentration, and inefficient load transfer, limiting the mechanical benefits that could otherwise be achieved.^22^ Surface functionalization strategies have therefore been explored to improve dispersion and interfacial adhesion, including noncovalent adsorption of surfactants and covalent attachment of organic moieties.^23^ Among these, polymer grafting is especially attractive, as it allows the inorganic nanostructure to be chemically integrated into the polymer matrix while preserving the intrinsic properties of both components.^24,25^

Importantly, covalent polymer grafting offers an additional, largely unexplored advantage in the context of WS_2_-based composites: the possibility to selectively passivate reactive edge and defect sites. By anchoring polymer chains at chemically active locations, direct contact between the polymer melt and catalytically active WS_2_ sites may be suppressed, potentially preventing undesirable chemical reactions during processing. However, this hypothesis has not yet been experimentally validated, and the role of WS_2_ edge chemistry in governing melt stability and processability remains essentially unexplored.

Despite extensive interest in WS_2_ as a reinforcing and functional nanofiller, its chemical reactivity toward polymer melts has remained essentially unexplored. In particular, no direct experimental evidence has been reported linking the edge chemistry of WS_2_ to polymer degradation phenomena under melt-processing conditions, even though WS_2_ edge and defect sites are known to catalyze oxygen-based reactions in heterogeneous catalysis.^26^ In this work, we address this gap by investigating the role of WS_2_ edge chemistry in melt-processed polymer composites and by proposing an edge- and defect-selective polymer-grafting strategy to stabilize WS_2_–polyester systems under extrusion conditions. WS_2_ nanopowders were selectively functionalized at edge and defect sites through covalent carboxylation and hydroxylation, followed by surface-initiated ring-opening polymerization of ε-caprolactone to yield polymer-grafted WS_2_ nanostructures with controlled grafting densities and chain lengths. The resulting hybrid nanomaterials were compounded into a PCL matrix and processed by LF-FGF, enabling a direct assessment of melt rheology, printability, molecular-weight stability, and mechanical performance in both in-plane and interlayer directions. By systematically comparing composites containing polymer-grafted and unmodified WS_2_, we demonstrate that pristine WS_2_ induces severe catalytic degradation of the polyester matrix during melt processing, whereas polymer grafting effectively passivates reactive edge and defect sites, preserves molecular weight, and enables mechanically robust, printable composites. Polyesters were deliberately selected in this study as chemically sensitive model systems, as ester bonds are known to be susceptible to both acid- and base-catalyzed scission under melt-processing conditions, allowing chemically driven effects to be unambiguously revealed. While the extent of degradation is therefore expected to depend on polymer chemistry, the presence of catalytically active edge and defect sites in layered TMDs is an intrinsic structural feature. As such, the underlying phenomenon of edge-mediated chemical activity may not be polyester-specific in origin, although its manifestation is demonstrated here for polyester systems. Beyond the specific WS_2_–PCL system investigated here, this study identifies edge-site passivation as a broadly applicable design principle to mitigate chemically driven polymer degradation during melt processing, particularly for layered nanomaterials with intrinsically reactive edge and defect sites.

## Results and Discussion

To enable the compatibilization of WS_2_ with thermoplastic polymer matrices, the pristine micropowder was subjected to a sequence of surface-modification steps designed to expose free hydroxyl groups for the subsequent SI-ROP of ε-caprolactone (Figure 1). The key challenge was to introduce organic functional groups through a truly covalent interaction with the inorganic surface while preserving the structural integrity of the WS_2_ phase. For this purpose, we adapted the polycarboxylation strategy reported by Raichman et al., who achieved the covalent grafting of 2-bromoacetic acid onto WS_2_ nanotubes in anhydrous DMF using AgOAc, through a modified Vilsmeier–Haack–like mechanism (Figure S1).^27^ This reaction pathway affords −COOH functional groups stably anchored to WS_2_ and renders the material dispersible and derivatizable. Extending this chemistry from curved nanotubes to WS_2_ micropowders, which consist of multilayered nanosheets, required consideration of the inherent structural anisotropy of platelet-like TMDs. Whereas the cylindrical curvature of nanotubes exposes undercoordinated atoms across the entire surface, essentially turning the whole nanotube into an “edge,” the layered structure of WS_2_ micropowder creates a sharp distinction between the chemically inert basal planes and the highly reactive sheet edges. On mechanistic grounds, therefore, functionalization in the micropowder is expected to occur preferentially at the edges and defect-rich lateral domains rather than at the basal planes, whose S–W–S coordination environment remains saturated and unreactive.^29,30^ The resulting carboxylated nanomaterial (WS_2_–COOH) was then esterified with ethylene glycol under acid catalysis to yield hydroxylated WS_2_–OH, which is an effective macroinitiator for ROP.

**Figure 1:**
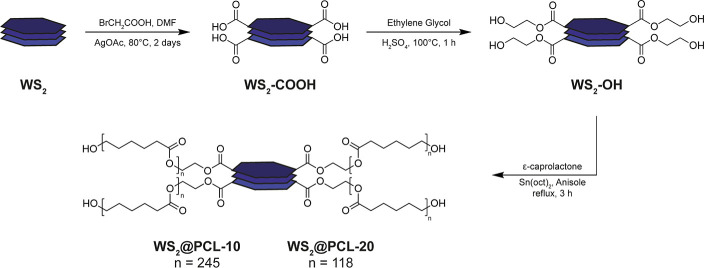
Strategy for the edge- and defect-selective functionalization and polymer grafting of WS_2_ nanopowder.

XPS analysis confirms that the WS_2_ lattice remains chemically intact throughout the carboxylation and esterification steps (Figure S2 and Table S1). The W 4f region displays the characteristic W^4+^ doublet at 32.4 eV in all samples, and the relative abundance of W^6+^ species remains unchanged, indicating that the pre-existing surface WO_3_ layer is neither removed nor altered by the functionalization sequence. Likewise, the S^2–^ 2p doublet at 161.6 eV is preserved, demonstrating that the 2H-WS_2_ core structure is unaffected.

The most significant changes occur in sulfur- and oxygen-based surface contaminants. Pristine WS_2_ exhibits a clear S^6+^ contribution, together with a high-binding-energy O 1s component at 532.8 eV, both consistent with sulfate residues commonly found in commercial WS_2_ powders. After carboxylation (WS_2_–COOH), both signatures disappear completely, confirming quantitative removal of sulfate species. In parallel, the O 1s envelope changes markedly: the high-BE component vanishes and two new contributions arise, a pronounced low-BE peak at 529 eV and a smaller oxide-like component at 530.3 eV, consistent with the formation of carboxylated and oxygenated fragments at the surface. The carbon content increases slightly after both functionalization steps, which provides an additional, though modest, indication of organic groups being introduced. As expected for an edge- and defect-selective reaction occurring at low surface density, these changes remain subtle because the chemically modified edge and defect sites are strongly diluted by the overwhelming spectral contributions of the basal planes and by adventitious carbon.

XRD measurements (Figure S3) were performed to assess whether the functionalization steps alter the long-range ordering of the WS_2_ layers. All samples display the characteristic reflections of the 2H phase, with no additional peaks attributable to oxidation, heterophase formation, or structural degradation. Moderate peak broadening is observed for all samples, which is expected given the nanometric character of the layered WS_2_ platelets. Crystallite size values extracted through Lorentzian peak fits and Scherrer analysis (Table S2) reveal a pronounced anisotropic trend: the (00L) reflections ((002), (004), (006)) exhibit large crystallite sizes in the 50–80 nm range, indicating long-range stacking coherence along the *c* axis that is characteristic of multilayer WS_2_. Crucially, no significant broadening of these reflections occurs upon either carboxylation or esterification, demonstrating that the layered structure remains fully preserved and that no exfoliation or thinning takes place during the chemical treatments. By contrast, the mixed-index reflections (103) and (105) yield much smaller crystallite sizes (13–17 nm), reflecting limited in-plane coherence, higher defect densities, and the presence of microstrain within the lateral domains. These features are typical of WS_2_ nanoplatelets and remain qualitatively unchanged after functionalization.^31^ Functionalization induces only a slight, systematic decrease in crystallite sizes across all hkl reflections, consistent with a minor increase in structural disorder that does not alter the overall multilayer slab architecture. The combined XRD and XPS results confirm that the functionalization sequence effectively introduces new surface moieties while leaving the underlying WS_2_ lattice structurally and chemically intact.

The hydroxylated WS_2_–OH nanostructures were employed as macroinitiators for the ROP of ε-caprolactone, performed in anisole under green solvent conditions, following procedures previously described in the literature.^32^ This approach was designed to generate polymer-grafted WS_2_ nanostructures that display enhanced dispersibility and interfacial compatibility within thermoplastic PCL matrices.

Two hybrid nanomaterials were prepared, denoted WS_2_@PCL-10 and WS_2_@PCL-20, where the numerical values represent the theoretical inorganic content (wt %) in the polymer–inorganic hybrids. Gravimetric analysis after alkaline hydrolysis of the grafted polymer chains, revealed effective inorganic contents of 10.3 ± 0.2 wt % and 19.9 ± 0.3 wt % for WS_2_@PCL-10 and WS_2_@PCL-20, respectively. The successful polymerization of the cyclic monomer was confirmed by ^1^H NMR (Figures 2a,b and S4) and FTIR (Figure 2c). Integration of the terminal monomer signals in the ^1^H NMR spectra yielded average molecular weights of (27.9 ± 2.8) kDa for WS_2_@PCL-10 and (13.5 ± 1.4) kDa for WS_2_@PCL-20. On the other hand, FTIR only shows the fingerprint of PCL macromolecules.

**Figure 2:**
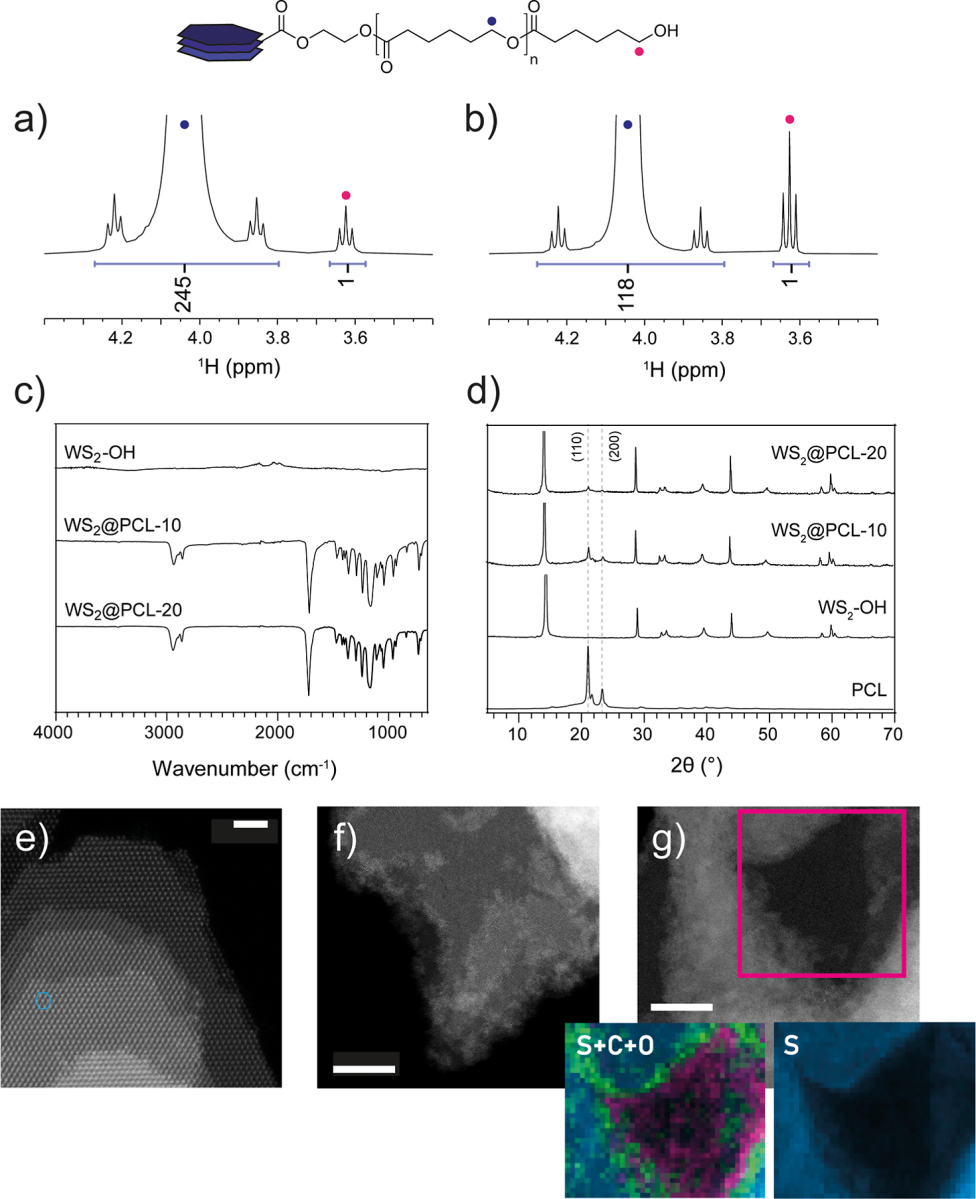
Characterization of polymer-grafted nanostructures. End-group ^1^H NMR region with relative peak integrals and spectral assignments for (a) WS_2_@PCL-10 and (b) WS_2_@PCL-20. (c) ATR-FTIR of WS_2_–OH, WS_2_@PCL-10 and WS_2_@PCL-20. (d) Experimental X-ray diffractograms of PCL, WS_2_@PCL-10 and WS_2_@PCL-20 together with the (hkl) assignment of the main peaks of PCL. (e) Atomic-resolution HAADF-STEM image of pristine commercial WS_2_ showing sharp, atomically clean step edges. Scale bar = 2 nm. (f) HAADF-STEM image of the polymer-grafted hybrid, WS_2_@PCL-20, revealing a diffuse, low-contrast halo at the step edges attributed to semicrystalline PCL chains. Scale bar = 10 nm (g) HAADF-STEM image and corresponding EELS elemental mapping (S, C, O). Where localized enrichment of the C and O signals at the WS_2_ terrace terminations provides direct evidence for edge- and defect-selective polymer grafting. Scale bar = 10 nm.

TGA (Figure S5) shows that WS_2_ undergoes only minimal mass loss (below 5 wt % of the initial weight), most likely due to the presence of impurities such as tungsten oxides or other residues typically found in commercial WS_2_. As expected, neat PCL fully decomposes by 600 °C. Considering these reference profiles, the residual masses of the composites confirm PCL contents of approximately 20 wt % for WS_2_@PCL-20and 10 wt % for WS_2_@PCL-10, consistent with the targeted compositions. Furthermore, the onset of degradation for the grafted PCL fraction in WS_2_@PCL-20occurs at a lower temperature than in WS_2_@PCL-10, and both degrade at temperatures lower than neat PCL. This trend suggests that the PCL chains grafted onto the WS_2_ surface are shorter than those of the PCL matrix, in agreement with the ^1^H NMR results.

Combining the grafted-chain molecular weights with the measured inorganic contents allows calculation of the number of PCL chains per gram of WS_2_, which corresponds to the density of hydroxyl initiating sites on WS_2_–OH. These values amount to (3.1 ± 0.3) × 10^–4^ mol/g for WS_2_@PCL-10 and (3.0 ± 0.3) × 10^–4^ mol/g for WS_2_@PCL-20, demonstrating that both systems possess nearly identical densities of grafting sites. Such close similarity strongly supports the interpretation that functionalization is confined to edge terminations and defect-associated oxygenated sites, consistent with the minimally exfoliated nature of the WS_2_ platelets and the structural information obtained from XRD, as well as the absence of nongrafted PCL chains.

XRD patterns of WS_2_@PCL-10 and WS_2_@PCL-20 (Figure 2d) feature the characteristic 2H-WS_2_ reflections superimposed upon the two well-defined PCL crystalline peaks at 21.5° and 23.7°, which correspond to the (110) and (200) planes. This confirms that PCL maintains its intrinsic semicrystalline structure within the hybrid nanomaterials and that the grafting procedure neither disrupts polymer crystallinity nor alters the ordering of the inorganic core. Scherrer analysis of the WS_2_ reflections (Table S3) reveals crystallite size values that mirror those of the WS_2_–OH precursor, further demonstrating that polymer grafting does not influence the multilayer nature of the WS_2_ nanostructures. Together, these results validate that the grafting strategy produces organic–inorganic hybrids in which both components retain their distinct structural features.

To complement the bulk averaged structural data obtained via XRD and spectroscopy, HAADF-STEM was employed to provide direct local visualization of the hybrid nanostructures and verify the spatial distribution of the grafted polymer. Figure 2e displays a representative atomic-resolution HAADF image of the pristine commercial WS_2_. Since HAADF signal intensity is directly proportional to sample thickness and atomic number (Z-contrast), the material exhibits a characteristic terrace-like topography where individual basal planes are clearly distinguishable by discrete changes in contrast intensity. The step edges of the pristine material appear atomically sharp and clean, indicating well-crystallized terminations with no amorphous residue, consistent with the high crystallinity observed in XRD. In contrast, the morphology of the polymer-grafted analogue, WS_2_@PCL-20, reveals distinct interfacial features (Figure 2f). While the multilayered, terrace-like architecture is preserved (corroborating the XRD findings that the functionalization process does not induce exfoliation or structural degradation of the inorganic core) the step edges differ markedly from those of the pristine material. Instead of the abrupt atomic termination seen in the unmodified WS_2_, the edges of WS_2_@PCL-20 are decorated with a diffuse, lower-contrast halo. This feature is attributed to the presence of the semicrystalline grafted PCL chains. Notably, no continuous amorphous polymer layer is observed on the basal-plane surfaces, which remain atomically sharp and well-defined. Importantly, despite the superimposition of this organic adlayer, the underlying crystalline lattice of the WS_2_ edges remains well-defined, further confirming that the chemical functionalization passivates the active sites without eroding the inorganic framework.

To unequivocally assign this contrast to the grafted polyester chains, EELS mapping was performed (Figure 2g). The elemental maps reveal a clear spatial correlation between the inorganic and organic components. The sulfur (S) signal clearly delineates the WS_2_ terraces. Crucially, the combined maps for carbon (C) and oxygen (O) show a specific enrichment along the step edges of the WS_2_ sheets. While a background carbon signal is present due to the holey carbon support grid, the localized intensification of the C and O signals precisely at the terrace terminations provides compelling evidence of edge- and defect-selective grafting. This direct morphological observation is in excellent agreement with the XPS data, which showed the emergence of organic oxygen species, and supports the mechanistic hypothesis that PCL chains are covalently grown from the reactive edge and defect sites of the WS_2_ platelets.

The synthesized WS_2_@PCL nanostructures were compounded into thermoplastic PCL via twin-screw extrusion to obtain composite formulations containing 0.2, 0.5, and 1.0 wt % of inorganic filler. In all cases, the nanostructures dispersed uniformly throughout the polymer matrix, and no macroscopic agglomerates were visually detected. MFR measurements were performed to evaluate the influence of the nanofillers on melt viscosity (Figure 3a and Table 1). For composites containing WS_2_@PCL, the MFR values remained comparable to those of neat PCL, with only moderate increases at higher filler loadings. In stark contrast, composites prepared with unmodified WS_2_ displayed a dramatic increase in MFR relative to neat PCL: at only 0.2 wt % loading, the MFR increased to 23.7 ± 1.1 g/10 min, and at 1.0 wt % reached 49.5 ± 3.6 g/10 min. This indicates severe reductions in melt viscosity and melt strength.

**Figure 3:**
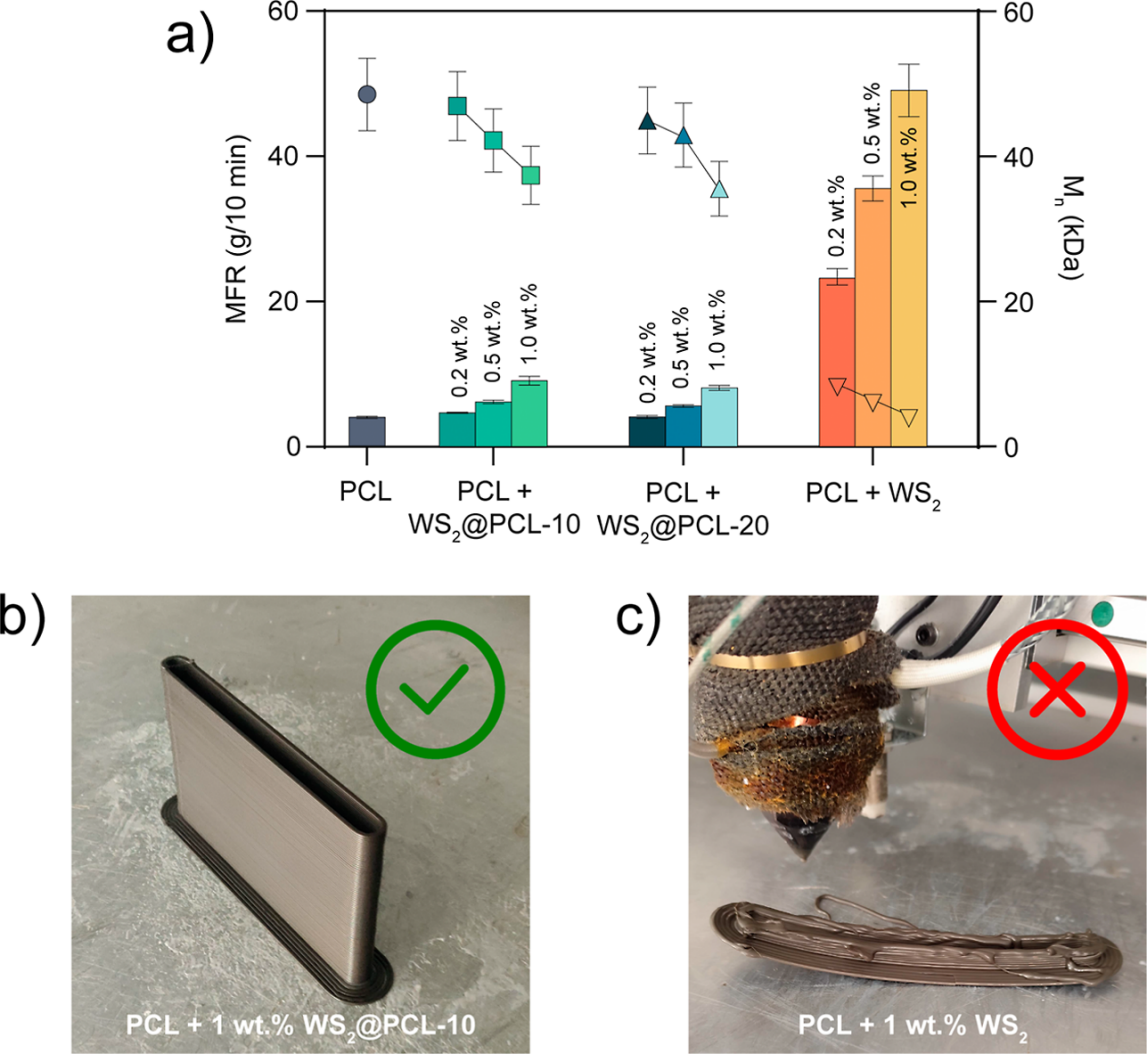
(a) Melt flow rate (bars) and average molecular weight (points) of pristine PCL and WS_2_-loaded PCL composites. Plotted data are reported in (Table S4 b,c) Comparison between the LF-FGF 3D printing process for modified and unmodified WS_2_ nanocomposites.

**Table 1:** Composition of the Prepared WS_2_ Nanocomposites

	blank PCL	WS_2_@PCL	WS_2_ content
PCL	100 wt %	-	-
PCL + 0.2% WS2@PCL-10	98 wt %	2 wt %	0.2 wt %
PCL + 0.5% WS2@PCL-10	95 wt %	5 wt %	0.5 wt %
PCL + 1.0% WS2@PCL-10	90 wt %	10 wt %	1.0 wt %
PCL + 0.2% WS2@PCL-20	99 wt %	1 wt %	0.2 wt %
PCL + 0.5% WS2@PCL-20	97.5 wt %	2.5 wt %	0.5 wt %
PCL + 1.0% WS2@PCL-20	95 wt %	5 wt %	1.0 wt %
PCL + 0.2% WS_2_	99.8 wt %	-	0.2 wt %
PCL + 0.5% WS_2_	99.5 wt %	-	0.5 wt %
PCL + 1.0% WS_2_	99 wt %	-	1.0 wt %

These rheological differences translated directly into the printability of the composites during LF-FGF. Neat PCL and WS_2_@PCL-containing composites (up to 1.0 wt %) could be printed with little to no defects in both the horizontal (XY) and vertical (XZ) planes (Figures 3b and S6). By contrast, even low loadings of unmodified WS_2_ produced highly distorted, brittle, and unstable structures, with continuous printing becoming impossible at 1.0 wt % filler content (Figure 3c). In particular, the MFR reaches values of approximately 50 g/10 min for PCL + 1.0 wt % WS_2_, which is found too high for polymer processing by LF-FGF.^33^

The dramatic increase in melt flow rate observed for composites containing pristine WS_2_ provides the first indication that WS_2_ is not chemically inert under melt-processing conditions. At filler loadings as low as 0.2 wt %, the MFR increases by more than *a* factor of 5 relative to neat PCL and reaches values incompatible with stable extrusion at higher loadings. Such pronounced rheological changes cannot be explained by physical dispersion effects alone and instead point to a chemically driven reduction of polymer molecular weight. To directly probe this hypothesis, the molecular weight of the PCL matrix after extrusion was quantified by ^1^H NMR end-group analysis (Figures S7 and S8). For this purpose, the extruded composites were analyzed without prior separation of the inorganic filler, exploiting the complete solubility of PCL and the NMR silence of WS_2_. The results are striking (Figure 3a): extrusion in the presence of pristine WS_2_ induces a nearly 10-fold decrease in the number-average molecular weight of PCL.

To our knowledge, this constitutes the first direct experimental evidence that WS_2_ nanopowders can strongly promote polyester chain scission under melt-processing conditions through a surface-mediated process. The apparent absence of similar degradation effects in previous reports on WS_2_-based polymer composites likely arises from a combination of polymer chemistry, processing conditions, and the metrics typically used to evaluate composite performance. Susceptibility to chemically driven degradation is strongly polymer-dependent: polyesters such as PCL contain ester linkages that are particularly sensitive to catalytic scission, whereas many earlier WS_2_ composite studies focus on polymers with more chemically robust backbones (e.g., polyolefins or aromatic thermoplastics), where analogous effects may be less pronounced.^34–36^ In addition, degradation is highly dependent on processing conditions such as temperature, residence time, and filler dispersion, which may not always be sufficient to trigger measurable molecular-weight changes. Finally, most previous studies primarily assess mechanical, tribological, or morphological properties of melt-processed composites without direct evaluation of polymer molecular weight or melt rheology. As demonstrated here, substantial chain scission can occur even at low WS_2_ loadings and may therefore remain undetected if molecular-weight analysis is not performed.

To clarify the origin of the observed degradation, several common degradation pathways were evaluated against the experimental observations. Thermo-oxidative degradation is unlikely, as all materials were processed under identical thermal and shear histories at relatively mild temperatures for PCL (≤120 °C), yet significant molecular-weight reduction occurs only in the presence of pristine WS_2_. Neat PCL and composites containing polymer-grafted WS_2_ show only minor molecular-weight changes typical of melt processing. Acid-catalyzed hydrolysis is also improbable, since all materials were thoroughly dried prior to processing and no aqueous phase is present during extrusion. Moreover, the strong suppression of molecular-weight loss after polymer grafting, while maintaining identical processing conditions, indicates passivation of specific reactive surface sites rather than neutralization of a bulk acidic environment. Generic filler-induced or shear-driven degradation can likewise be excluded, as pristine and polymer-grafted WS_2_ composites were processed under identical conditions and differ only in filler surface chemistry. Together, these observations indicate that the degradation is associated with chemically active sites of the WS_2_ surface. Although direct identification of the active sites is beyond the scope of this study, the combined evidence supports an edge-mediated catalytic process.

The grafting strategy provides additional insight into the origin of the degradation. The PCL chains grown from the WS_2_ surface have number-average molecular weights of (27.9 ± 2.8) kDa and (13.5 ± 1.4) kDa, compared to (50.6 ± 5.1) kDa for the commercial PCL matrix. Although shorter than the matrix chains, these values correspond to oligomeric-to-polymeric species capable of entanglement rather than low-molecular-weight plasticizers. Importantly, the presence of grafted PCL chains cannot account for the nearly order-of-magnitude reduction in matrix molecular weight observed when pristine WS_2_ is used. The drastic molecular-weight collapse induced by unmodified WS_2_ therefore cannot be rationalized in terms of polymer–polymer miscibility or dilution effects. Instead, the results indicate a chemically driven degradation process that is effectively suppressed upon passivation of the reactive surface sites by grafted polymer chains.

Identification of the specific low-molecular-weight degradation products could provide additional mechanistic insight. However, such analysis is beyond the scope of the present study, which focuses on establishing the processing-relevant consequences of WS_2_-induced chain scission and its suppression via surface passivation.

To assess whether the molecular-weight changes induced by melt processing evolve during storage, additional aging studies were conducted on selected samples. Extruded pellets were stored under ambient laboratory conditions and reanalyzed after 12 months using the same end-group ^1^H NMR methodology employed for the freshly processed materials. Within experimental uncertainty, no significant changes in number-average molecular weight or detectable alterations in the polymer structure were observed during storage (Table S4). These results indicate that the chain scission reported in this work occurs primarily during the melt-processing step and does not continue under ambient storage conditions. This further supports that the degradation mechanism is process-induced rather than time-dependent.

Tensile tests were performed on specimens extracted from XY- and XZ-printed plates (Figures 4, S9 and Table S5). Across all formulations, the incorporation of WS_2_-based nanofillers results in a consistent reinforcement of the polymer matrix. In the XY direction, the Young’s modulus of neat PCL (245 ± 8 MPa) increases markedly upon addition of the modified fillers, reaching 341 ± 7 MPa for 1.0 wt % WS_2_@PCL-10, (+39%), and 313 ± 8 MPa for 1.0 wt % WS_2_@PCL-20 (+28%). A comparable trend is observed in XZ, where neat PCL (277 ± 13 MPa) rises to 402 ± 12 MPa with WS_2_@PCL-10 at 1.0 wt % (+45%) and 384 ± 6 MPa with WS_2_@PCL-20 (+39%). These improvements scale monotonically with filler content and are systematically greater for WS_2_@PCL-10, consistent with the longer grafted PCL chains providing more effective interfacial entanglement and load transfer.

**Figure 4:**
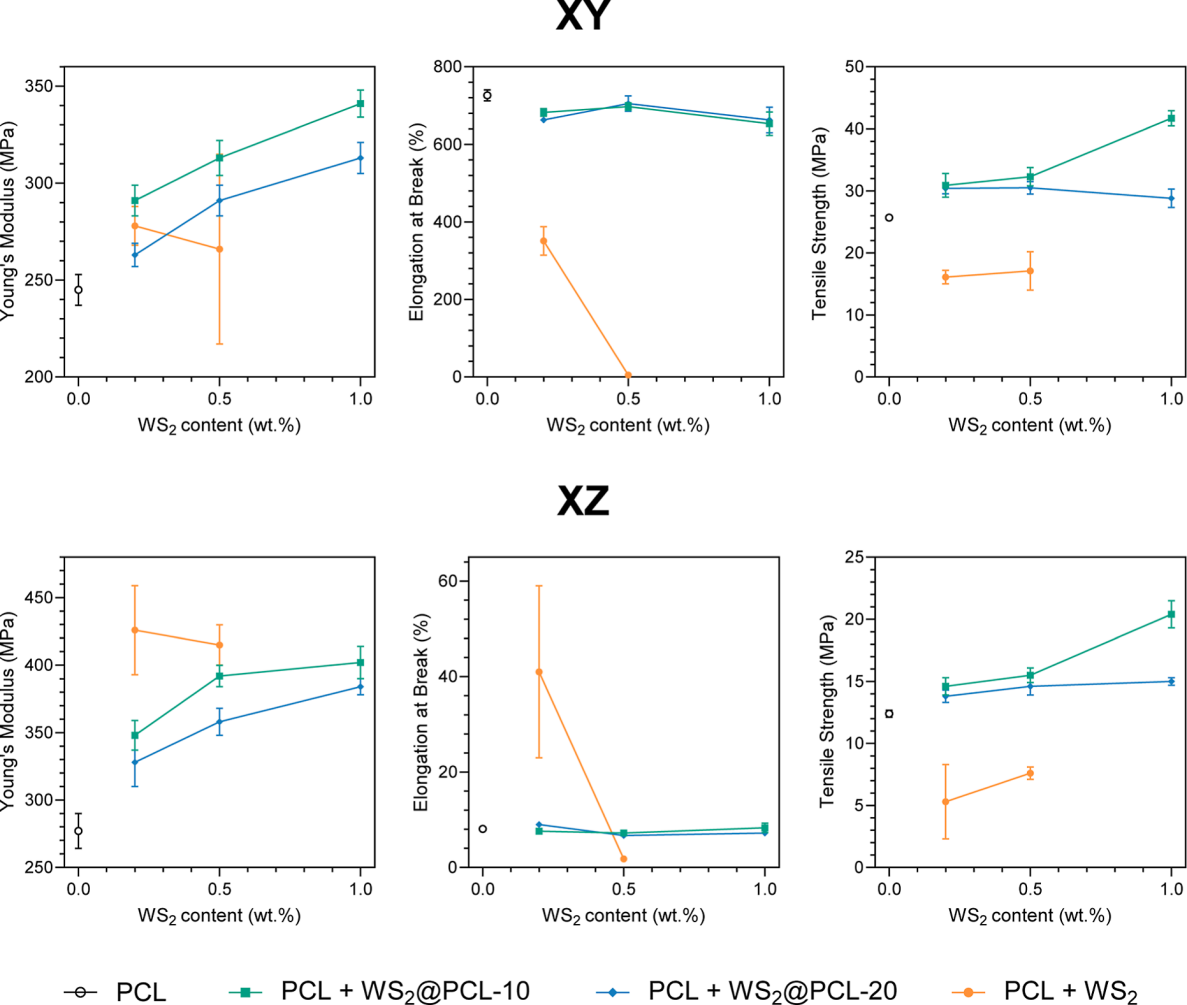
Tensile properties of 3D printed materials.

Crucially, the modified nanofillers do not compromise the ductility of the printed materials. In XY, the elongation at break of PCL (726 ± 14%) remains essentially unchanged: WS_2_@PCL-10 gives 682 ± 10% at 0.2 wt % and 653 ± 30% at 1.0 wt %, while WS_2_@PCL-20 ranges from 705 ± 20% to 663 ± 33% across the same loadings. A similar invariance is observed in XZ, where the elongation at break of neat PCL (8.1 ± 0.7%) remains between 7.2 ± 0.2% and 9.0 ± 0.2% for all modified formulations. The narrow error bars associated with these samples further highlight their microstructural stability, in contrast with the high variability seen in composites containing unmodified WS_2_. Improved interfacial compatibility provided by grafted PCL chains likely contributes to the enhanced mechanical performance and reduced data scatter observed in the printed composites, but this effect is secondary to the chemical stabilization of the polymer melt.

Tensile strength is also strongly enhanced by the modified fillers. In XY, the strength at break increases from 25.7 ± 0.3 MPa for neat PCL to 41.7 ± 1.2 MPa for 1.0 wt % WS_2_@PCL-10 (+62%). In XZ, the improvement is similarly pronounced, rising from 12.4 ± 0.3 MPa to 20.4 ± 1.1 MPa at the same loading (+65%). WS_2_@PCL-20 also improves tensile performance, though less markedly, again reflecting the dominant role of chain length in mediating stress transfer across the filler–matrix interface. The monotonic increase of both modulus and strength with filler content further confirms the excellent compatibility between PCL and the grafted nanostructures.

The behavior of unmodified WS_2_ contrasts sharply with that of the modified fillers. Although stiffness is increased (e.g., up to +54% in XZ at 0.2 wt %), this occurs at the expense of virtually all other mechanical properties. In XY, strain at break collapses from 726 ± 14% to 351 ± 37% at 0.2 wt % (-52%), and falls to only 5.2 ± 1.0% at 0.5 wt %, a dramatic 99% loss of ductility. Tensile strength also decreases substantially, dropping from 25.7 ± 0.3 MPa to 16.1 ± 1.1 MPa in XY and from 12.4 ± 0.3 MPa to 5.3 ± 3.0 MPa in XZ.

An isolated deviation occurs in XZ at 0.2 wt % unmodified WS_2_, where the material exhibits an unexpected and highly significant increase in ductility: the elongation at break rises from 8.1 ± 0.7% in neat PCL to 41 ± 18%, corresponding to an approximate 5-fold increase. This pronounced gain in strain at break, accompanied by a 22% increase in tensile strength (from 12.4 ± 0.3 MPa to 15.1 ± 1.6 MPa), suggests that the detected molecular weight reduction perturbs the microstructure in a way that transiently enhances interlayer adhesion and deformation capacity in the *Z* direction. Although this effect is not technologically exploitable, given the concurrent collapse of mechanical properties in all other loading conditions, it reveals a mechanistic avenue worth exploring for the targeted modulation of interlayer cohesion in LF-FGF printing systems.

To identify whether structural changes in the filler or matrix contributed to the divergent mechanical responses, XRD was performed on the printed composites with the highest printable WS_2_ loading (Figure S10). All diffractograms displayed the characteristic reflections of both PCL and WS_2_, with no detectable shifts, intensity variations, or broadening attributable to exfoliation, layer thinning, or changes in polymer crystallinity. The similarity of the diffractograms across all samples indicates that neither structural changes in the WS_2_ fillers nor alterations in PCL crystallinity are responsible for the observed mechanical property variations. Instead, the differences arise from graft-dependent interfacial interactions.

Finally, DSC was performed on the composites with 1.0 wt % of inorganic nanofiller to evaluate its influence on the thermal behavior of the thermoplastic materials. Under slow cooling (standard protocol at 10 °C/min, Figure 5a), the presence of WS_2_ significantly increases the cold-crystallization temperature (*T*_cc_) of PCL, shifting from 19.7 °C in neat PCL to 39.9 °C for unmodified WS_2_, and to 35.1 and 35.3 °C for PCL@WS_2_-20 and PCL@WS_2_-10, respectively. The crystallization enthalpy also increases sharply for the composite containing unmodified WS_2_, while the composites with modified WS_2_ exhibit values similar to neat PCL. Excessive crystallization during cooling can lead to excessive shrinkage during printing, resulting in defective prints. Thus, the surface modification of WS_2_ clearly contributes to a PCL thermal behavior closer to that of the unfilled polymer.

**Figure 5:**
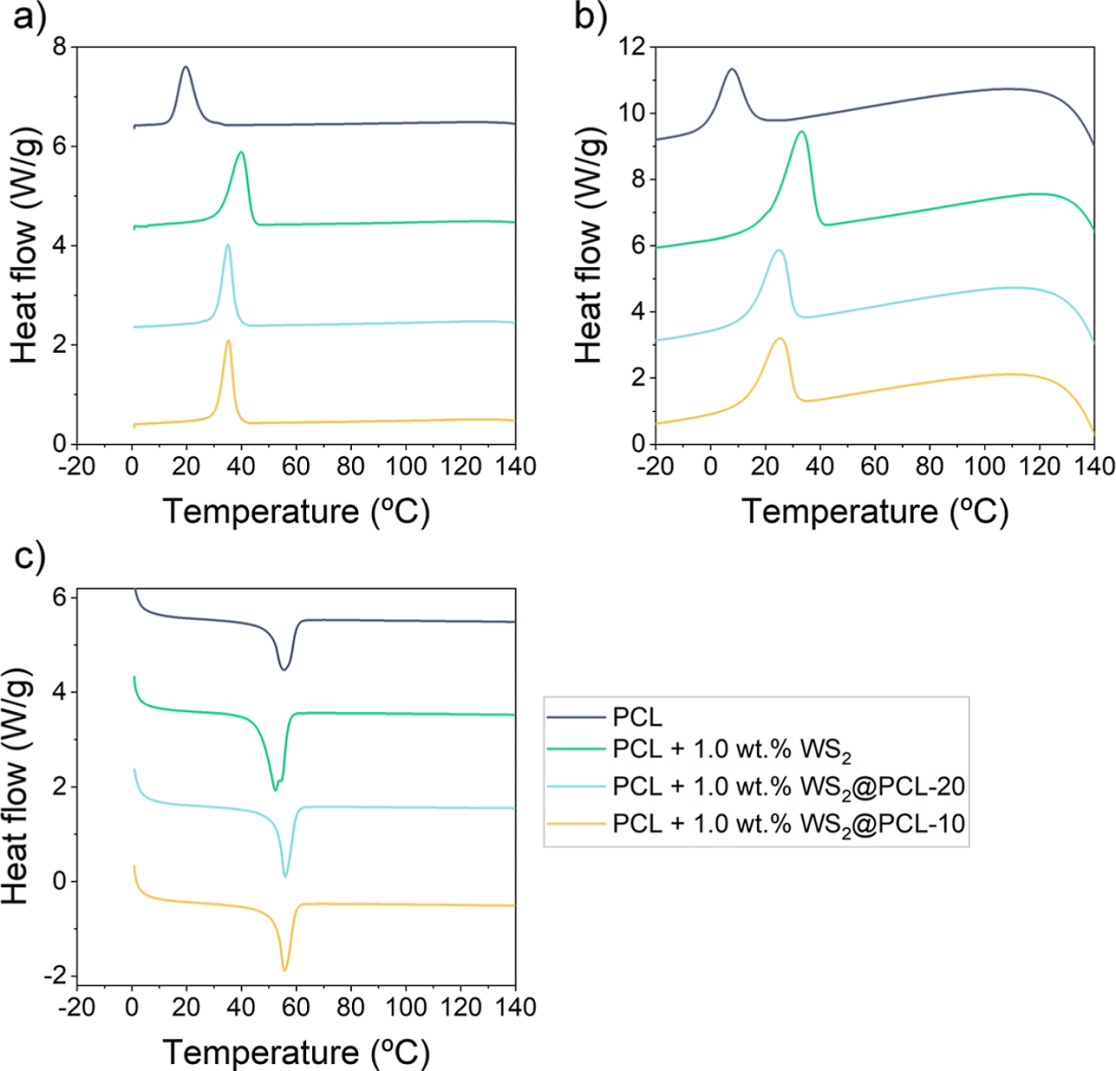
DSC thermograms of (a) cooling sweep at 10 °C/min; (b) cooling sweep at maximum speed starting at 50 °C/min at 150 °C and reaching 25 °C at 30 °C/min (see Figure S11 for more details), and (c) subsequent heating sweep.

This effect becomes more pronounced under rapid cooling (Figure 5b). This cooling is performed at the maximum speed available by the DSC instrument, following a previously optimized protocol that resembles the cooling of thermoplastic polymers in LF-FGF.^28^ In this case, the cooling starts from 150 °C at 50 °C/min °C and reaching 25 °C at 30 °C/min (see Figure S11 for more details). Although all samples start crystallizing at lower temperatures due to the faster cooling rate, the *T*_cc_ of the composite with unmodified WS_2_ is observed at 33 °C (above room temperature) indicating that crystallization can still occur during printing. In contrast, the PCL@WS_2_-20 and PCL@WS_2_-10 composites exhibit a *T*_cc_ at 24.5 and 24.8 °C, respectively, suggesting that even under fast cooling conditions representative of FGF-based printing, the degree of crystallization (and therefore shrinkage) during printing is reduced.

The subsequent heating scans (Figure 5c) show that the presence of WS_2_, either modified or unmodified, has only a minimal influence on the melting temperature (*T*_m_) of PCL, which remains near 56 °C. For the composite with unmodified WS_2_, *T*_m_ is slightly lower and the melting enthalpy is higher, which may be attributed to the previously discussed chain scission of PCL by WS_2_, since shorter polymer chains tend to melt earlier, just as they crystallize earlier during cooling. All numerical values of *T*_cc_, *T*_m_ and their corresponding enthalpies can be found in Table S6.

To prove whether the degradation phenomenon observed for the PCL/WS_2_ system is restricted to this specific material pair, additional experiments were performed using PLA as an alternative polyester matrix and MoS_2_ as a related transition metal dichalcogenide. PLA was melt processed under comparable extrusion conditions in the absence and presence of WS_2_ or MoS_2_ nanopowders, and the polymer molecular weight was evaluated by end-group ^1^H NMR analysis. As shown in Figure 6 exhibits a clear decrease in molecular weight after melt processing in the presence of both WS_2_ and MoS_2_, compared to neat PLA processed under identical conditions, indicating that chain scission also occurs in this system. However, the reduction in molecular weight is less pronounced than in the PCL/WS_2_ system. This behavior is consistent with the lower susceptibility of PLA to ester bond cleavage, likely due to the steric hindrance introduced by the methyl substituent, which reduces accessibility to the ester linkage compared to PCL.^37,38^

**Figure 6:**
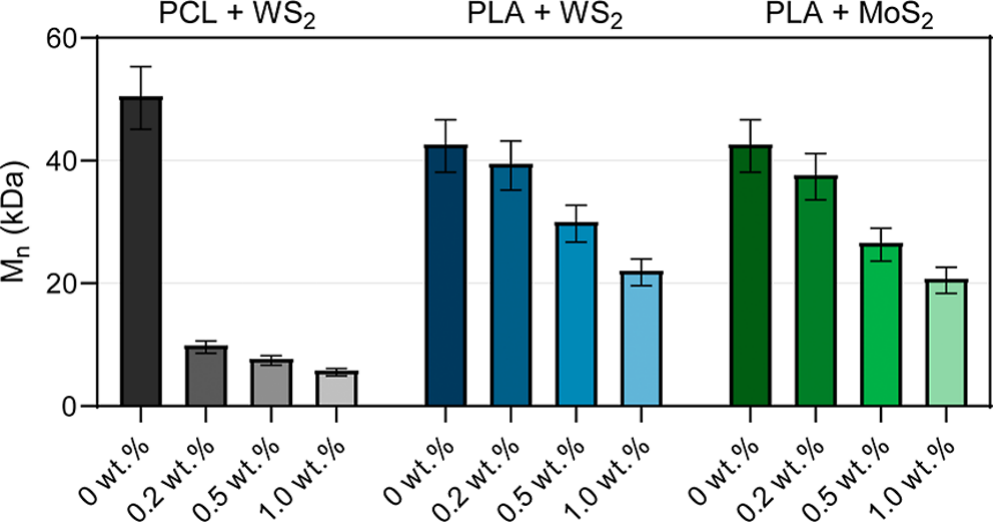
Number-average molecular weight (*M*_n_) of PCL and PLA after melt processing in the presence of transition metal dichalcogenide (TMD) nanopowders. Samples were processed under identical extrusion conditions with increasing filler loadings (0–1.0 wt %) of WS_2_ or MoS_2_, and *M*_n_ was determined by end-group ^1^H NMR analysis.

These observations indicate that the degradation phenomenon is not restricted to a single polymer–filler pair and can occur in different polyester matrices and with different TMDs, although its magnitude strongly depends on the polymer structure. Together, these results support the hypothesis that chemically active surface sites of TMD nanostructures can promote polyester chain scission under melt-processing conditions. While the present experiments provide initial evidence of this behavior, further work is underway to explore these systems in greater depth, including the role of surface functionalization and structure–property relationships.

## Conclusions

This work demonstrates that the successful integration of WS_2_ nanopowders into melt-processed thermoplastic matrices critically depends on controlling the chemical activity of their edge and defect sites. Through a sequential carboxylation–hydroxylation strategy followed by surface-initiated ring-opening polymerization of ε-CL, WS_2_ nanoplatelets were selectively functionalized at their edges while preserving their crystal structure. The resulting polymer-grafted nanostructures disperse homogeneously in PCL and enable the fabrication of mechanically robust components by LF-FGF, exhibiting increased stiffness and tensile strength while maintaining ductility and low data scatter.

End-group ^1^H NMR analysis reveals that pristine WS_2_ strongly promotes polyester chain scission during melt processing, leading to a dramatic reduction in polymer molecular weight. This degradation manifests macroscopically as reduced melt viscosity, poor printability, and brittle failure of printed parts. In contrast, grafting polymer chains onto WS_2_ effectively suppresses this behavior by passivating chemically active surface sites, enabling stable processing and high-performance composites.

More broadly, these findings challenge the common assumption that layered nanomaterials behave as chemically inert fillers during melt processing. Instead, exposed edge and defect sites of WS_2_ nanopowders exhibit pronounced catalytic activity toward polyester degradation. While PCL represents a particularly sensitive matrix due to the lability of ester bonds, preliminary experiments performed with another polyester (PLA) and a related dichalcogenide (MoS_2_) indicate that the phenomenon is not restricted to the specific PCL/WS_2_ system. Together, these results highlight the critical role of nanofiller surface chemistry in governing polymer stability during melt processing and demonstrate that selective edge-site passivation provides an effective strategy for incorporating chemically active layered nanomaterials into thermoplastic matrices and additive manufacturing processes.

## Experimental Section

If not otherwise specified, all reagents and solvents were purchased from Sigma-Aldrich (St. Louis, MO, USA) and used as received. ε-Caprolactone (ε-CL) was dried over CaH_2_ and distilled under vacuum prior to use. Thermoplastic telechelic PCL matrix (eSUN600C, Mw = 65000 g/mol) was purchased from eSun (Shenzen, China).

### Synthesis of Surface-Carboxylated WS_2_ Nanosheet (WS_2_–COOH)

Surface carboxylation of WS_2_ was adapted from a procedure previously reported by Raichman et al. in 2015 on WS_2_ nanotubes.^27^ Typically, 50 g of bromoacetic acid are dissolved in 150 mL of dry DMF under Ar atmosphere in a 500 mL round bottomed flask, followed by the addition of 500 mg of silver­(I) acetate. Then, 10 g of WS_2_ micropowder were added, and the mixture was placed in a sonicating bath under inert atmosphere for 2 h to ensure homogeneous dispersion. Then, the flask was placed in an oil bath at 80 °C for 24 h under vigorous stirring followed by 2 h of sonication and further 24 h in the oil bath. Finally, the mixture was cooled to room temperature, diluted with isopropanol and centrifugated (10 min at 4000 rpm) followed by repeated redispersion and recentrifugation in isopropanol (3 times). The obtained WS_2_–COOH was dried overnight in an oven at 80 °C. Yield = 95%.

### Synthesis of Surface-Hydroxylated WS_2_ Nanosheet (WS_2_–OH)

WS_2_–COOH from the previous step (9.5 g) was placed in a 500 mL round-bottomed flask equipped with magnetic stirrer and dropping funnel under Ar atmosphere and dispersed in 200 mL of ethylene glycol first by magnetic stirring, and by sonication in ultrasound bath (30 min). Then, 10 mL of 98% sulfuric acid were slowly added dropwise under vigorous stirring, and the mixture was kept at 100 °C for 1.5 h to ensure efficient esterification. Then, the mixture was cooled to room temperature, poured in a beaker and neutralized by slowly adding a saturated NaHCO_3_ solution until bubbling stopped occurring. WS_2_–OH was first isolated by centrifugation (10 min at 4000 rpm), then washed with water (twice) and finally with acetone (twice). The product was dried in an oven at 80 °C overnight. Yield = 96%.

### Surface-Initiated Ring Opening Polymerization (SI-ROP) of ε-CL on WS_2_ Surface

In a flame-dried 1 L round bottomed flask, WS_2_–OH (4.75 g for WS_2_@PCL-10 or 9.5 g for WS_2_@PCL-20) was dispersed in 120 mL of dry anisole containing 40 mL of freshly distilled ε-CL under Ar atmosphere. Then, the mixture was sonicated for 30 min in an ultrasound bath, then 1.15 mL of Sn­(oct)_2_ were added, and the mixture was stirred under reflux for 3 h. Then, the mixture was cooled to room temperature and poured in an excess of isopropanol under vigorous stirring to induce the precipitation of the polymer-grafted nanostructure. The mixture was then filtered and redissolved twice in dichloromethane followed by precipitation in isopropanol. Yield = 88% for WS_2_@PCL-20 and 84% for WS_2_@PCL-10.

### Evaluation of WS_2_ Content in WS_2_@PCL Masterbatches

The precise WS_2_ content in the WS_2_@PCL masterbatches was evaluated gravimetrically. A small piece of WS_2_@PCL (629 mg for WS_2_@PCL-10 and 347 mg for WS_2_@PCL-20) was placed in a vial followed by the addition of 5 mL of 3 M NaOH in H_2_O/MeOH 1:1 and boiled under vigorous stirring for 15 min. The alkaline hydrolysis led to the total depolymerization of ester bonds, leading to an aqueous dispersion of WS_2_–COO^–^. The mixture was then centrifugated, washed twice with water then with acetone, and the obtained solid was dried in an oven at 80 °C and weighed.

### Compounding of WS_2_@PCL Masterbatches in PCL Matrix

PCL, WS_2_, WS_2_@PCL-10 and WS_2_@PCL-20 were dried for at least 6 h at 40 °C in a vacuum oven to remove any residual moisture. Then, neat PCL with different filler contents (Table 1) were processed in a Scamex Rheoscam D20 (Isques, France) working at 72 rpm with a L/D of 47, and a temperature profile from the feeding hopper to the nozzle of 80–100–100–120–100 °C was used for the preparation of all the composites.

To examine whether the observations obtained for the PCL/WS_2_ system could extend to other polyester matrices and related transition metal dichalcogenides, additional melt-processing experiments were performed using poly­(lactic acid) (PLA) as an alternative polymer matrix and molybdenum disulfide (MoS_2_) as a related layered nanomaterial. PLA (Smart Materials 3D, Jaen, Spain) was compounded with WS_2_ or MoS_2_ at 0.2, 0.5, and 1.0 wt % in a Scamex Rheoscam D20 (Isques, France) twin-screw extruder working at 72 rpm with a L/D of 47, and a temperature profile from the feeding hopper to the nozzle of 180–180–190–200–200 °C. Neat PLA processed under identical conditions was used as a reference material. In all cases, a continuous filament was obtained and cut into small pieces of 2–3 mm length using a Scamex pelletizer.

### LF-FGF of WS2/PCL Composites

The composites were then used as feedstock in an LF-FGF Discovery 3D Granza machine purchased from Bárcenas CNC (Ciudad Real, Spain). Two types of plates were printed in the XY and XZ directions, according to ISO/ASTM 52921.^25,28^ Both plates were printed using a bead width of 2 mm, layer height of 1 mm, and printing speeds of 50 mm/s for the XY plates and 10 mm/s for the XZ plates. A constant temperature profile of 75/80/85 °C was established for all the materials studied after a temperature optimization process. These temperatures correspond to the three heating zones of the extruder, the last one being the closest to the nozzle. In all cases, the platform temperature was set to 40 °C to ensure good adhesion of the first layer and avoid warping. Then, a LEKN­(C1) 3020 CNC Router Machine Kit CNC was used to cut the specimens for tensile testing out of the printed plates, according to ISO 527. A 1.5 mm diameter flat milling cutter with two cutting edges was used to machine the specimens at a speed of 5000 rpm and a feed rate of 200 mm/min.

### Chemical Characterization

^1^H NMR spectra were obtained at 298 K on a Bruker 400 MHz Nuclear Magnetic Resonance (NMR) spectrometer. In all recorded spectra, chemical shifts are reported in ppm of frequency relative to the residual solvent signals (^1^H: 7.26 ppm for CDCl_3_). For the calculation of molecular weight via NMR, the signals of the alcoholic CH_2_ of PCL were integrated for both the terminal (3.6 ppm) and repeating unit (3.95 ppm). By setting at 1 the integral value of the end-group peak, the average molecular weight *M*_n_ of the polymer was calculated according to eq 1

1 $$M_{n}=\left(A_{PCL}+1\right)\cdot 114$$

where *A*_PCL_ is integral value of CH_2_–O signal of the repeating unit. When the molecular weight of the PCL matrix was being evaluated, *M*_*n*_ was further multiplied by *a* factor 2, in order to account for the telechelic nature of the commercial polymer matrix. For PLA, the molecular weight was calculated analogously by comparing the integral area of the peak of CH–O repeating units with the CH–OH signal of the end-group peak.

The results of the molecular weight calculations are expressed with an estimated error of ± 10%.

Attenuated Total Reflectance – Fourier Transform Infrared (ATR-FTIR) analyses were performed with a Bruker Alpha spectrophotometer, equipped with a Universal ATR accessory. All spectra were recorded as an average of 32 scans (range 4000–400 cm^–1^ with a resolution of 4 cm^–1^).

X-ray diffraction (XRD) analysis was performed on a Bruker D8 Advance spectrometer equipped with a VÅNTEC-1 high-resolution detector. A nickel filter of 0.15418 mm was used to make monochromatic the Cu­(Kα) radiation. The acquisition region was 5° < 2 theta <80°, with steps of 0.1° and count of intensity every second.

X-ray photoelectron spectroscopy (XPS) was performed with a SPECS Phoibos 150 MCD spectroscope using a monochromatic Al Kα source (1486.6 eV). Survey scans and high-resolution spectra of W 4f, S 2p, O 1s, and C 1s regions were acquired under identical instrumental conditions, using a fixed analyzer pass energy and constant step size. Charge compensation was applied, and all spectra were calibrated by referencing the W 4f_7/2_ peak to 32.4 eV. A linear background was subtracted from each high-resolution region, and peak fitting was carried out using Gaussian profiles with physically constrained parameters. For W 4f and S 2p, spin–orbit splitting and area ratios were fixed to the expected values for the 4f and 2p doublets, while all other parameters (peak positions, widths, and amplitudes) were optimized within chemically reasonable limits. The O 1s and C 1s regions were fitted using one to three Gaussian components depending on the sample, without imposing chemical assignments beyond binding-energy consistency. Atomic composition was performed using the manufacturer’s relative sensitivity factors (RSF) for each core level, after summing the areas of all components belonging to the same element.

### Morphological Characterization

Transmission Electron Microscopy (TEM) analyses were performed using an aberration corrected FEI Titan Themis electron microscope working at 200 kV. Given the hybrid material sensitivity to electron beam irradiation, strict dose control was implemented. For this, the monochromator of the microscope was utilized to adjust the probe current, ensuring all acquisitions were performed at currents maintained below 0.03 nA to prevent structural degradation. The local structural features and elemental distribution of the samples were investigated using High-Angle Annular Dark-Field Scanning (HAADF-S) TEM and Electron Energy Loss Spectroscopy (EELS). EELS spectra were acquired using a Gatan Dual EELS Spectrometer working with a 2.5 mm aperture with an energy dispersion of 0.25 eV/ch and a calculated energy resolution (i.e., full width at half-maximum (fwhm) of the zero-loss peak) of 1 eV. A current of 50 pA and a pixel time of 0.15 s were used.

#### Materials Characterization

The melt flow rate (MFR) was measured using a Lonroy LR-A001-A machine (China) working at 80 °C and with a load of 5 kg. At least 3 independent measurements were performed to ensure the reproducibility of the results.

The mechanical characterization of the printed specimens was assessed by tensile testing in a Shimadzu AGS-X machine (Kyoto, Japan) using a constant speed of 50 mm/s for the specimens printed in the XY direction and 5 mm/s for the specimens printed in the XZ direction. At least 5 specimens of each material were tested in all cases, in agreement with ASTM D638. Data are expressed as mean ± standard deviation (SD).

Differential Scanning Calorimetry (DSC) measurements were carried out on a TA Instruments Q20 under a nitrogen atmosphere. Samples were first heated from room temperature to 150 °C at 10 °C/min to erase thermal history. From this point, two cooling protocols were performed in separate experiments: (i) a standard slow cooling scan at 10 °C/min down to 0 °C, and (ii) a rapid cooling scan designed to mimic the fast cooling occurring during 3D printing,^28^ using the maximum cooling rate achievable by the instrument (starting at approximately 50 °C/min from 150 °C). After each cooling step, a second heating scan was recorded from 0 to 150 °C at 10 °C/min. The thermal stability of the materials was examined by thermogravimetric analysis (TGA) in a Q50 (TA Instruments). Following a typical procedure, a temperature sweep from room temperature up to 600 °C was performed using a constant rate of 10 °C/min. All the TGA experiments were carried out under a constant nitrogen flow.

## Supplementary Material



## Notes

Supporting Information file includes XPS, XRD, NMR, DSC, and TGA data, pictures of 3D printing processes, and tensile properties of 3D printed nanocomposite materials (PDF)
