# Supplementary material for: Polymer-Grafted WS2 Nanocomposites: from Edge-Site Passivation to Melt-Stable Fused Granular Fabrication
Source: ACS Nano. 2026 Apr 21;20(17):12978–89. doi: 10.1021/acsnano.5c22326 (PMC13151055; doi:10.1021/acsnano.5c22326)
Supplement: Supplementary file 1 [file nn5c22326_si_001.pdf]

Polymer-Grafted WS_2 Nanocomposites: From Edge-Site Passivation to Melt-Stable Fused Granular Fabrication

Mirko Maturi[1], Alberto Sanz de Leon[1], Luisa M. Valencia[1,2], Sergio I. Molina[1], Miriam Herrera[1]*

[1] *Dpto. Ciencia de los Materiales, I. M. y Q. I., IMEYMAT, Facultad de Ciencias, Universidad de Cádiz, Campus Río San Pedro, s/n, 11510 Puerto Real, Cádiz, Spain*

[2] *Instituto de Ciencia de Materiales de Sevilla (CSIC-US), Americo Vespucio 49, Seville 41092, Spain*

SUPPORTING INFORMATION

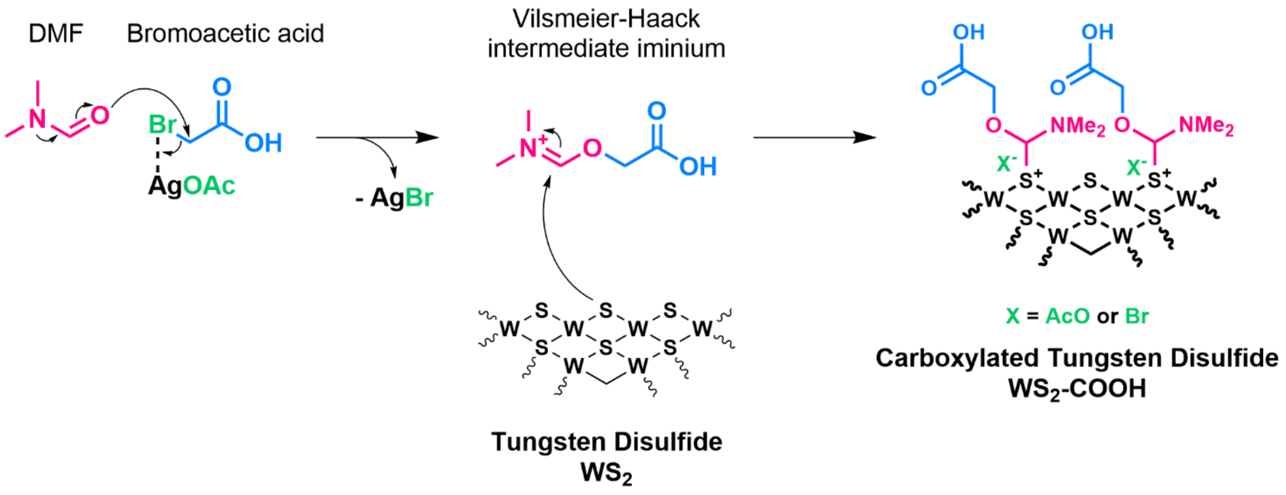

Figure S1. Mechanism proposed by Raichman et al. for the carboxylation of WS_2 using DMF and bromoacetic acid under Ag(I) catalysis. Adapted from ref. 24.

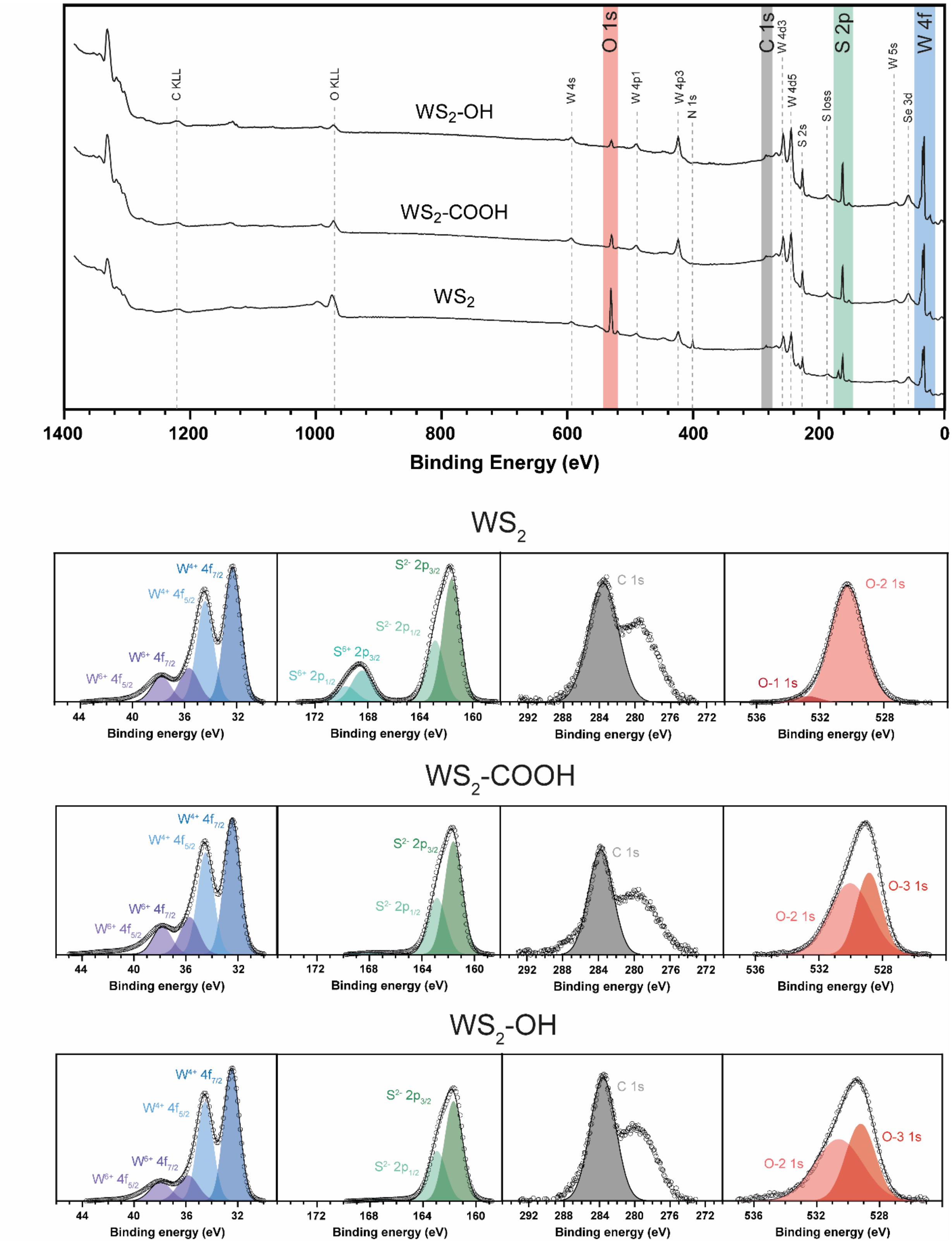

Figure S2. XPS analysis of WS_2 before and after the functionalization steps, together with the high-resolution spectra recorded in the W(4f), S(2p), C(1s) and O(1s) regions (dots), and their deconvolution (solid line) obtained as a sum of gaussian peaks (colored areas).

Table S1. Deconvolution of high-resolution XPS spectra. For each sample and for each chemical species, the deconvoluted peak position is reported, as well as the relative peak areas and the atomic composition calculated using the RSF provided by the spectrometer manufacturer.

	**WS_2**	**WS_2-COOH**	**WS_2-OH**
**Peak position**			
W^{4+}	32.4	32.4	32.4
W^{6+}	35.7	35.7	35.8
S^{2-}	161.6	161.6	161.7
S^{6+}	168.4	-	-
O-1	-	529.0	529.2
O-2	530.3	530.2	530.6
O-3	532.8	-	-
C	283.7	283.6	283.6
**Relative peak area**			
S^{6+}/S_{tot}	23%	-	-
W^{6+}/W_{tot}	20%	21%	16%
O-1/O_{tot}	-	41%	44%
O-2/O_{tot}	96%	59%	56%
**Atomic composition (mol.%)**			
**W**	16%	27%	25%
**S**	41%	54%	50%
**O**	37%	10%	11%
**C**	5%	9%	14%

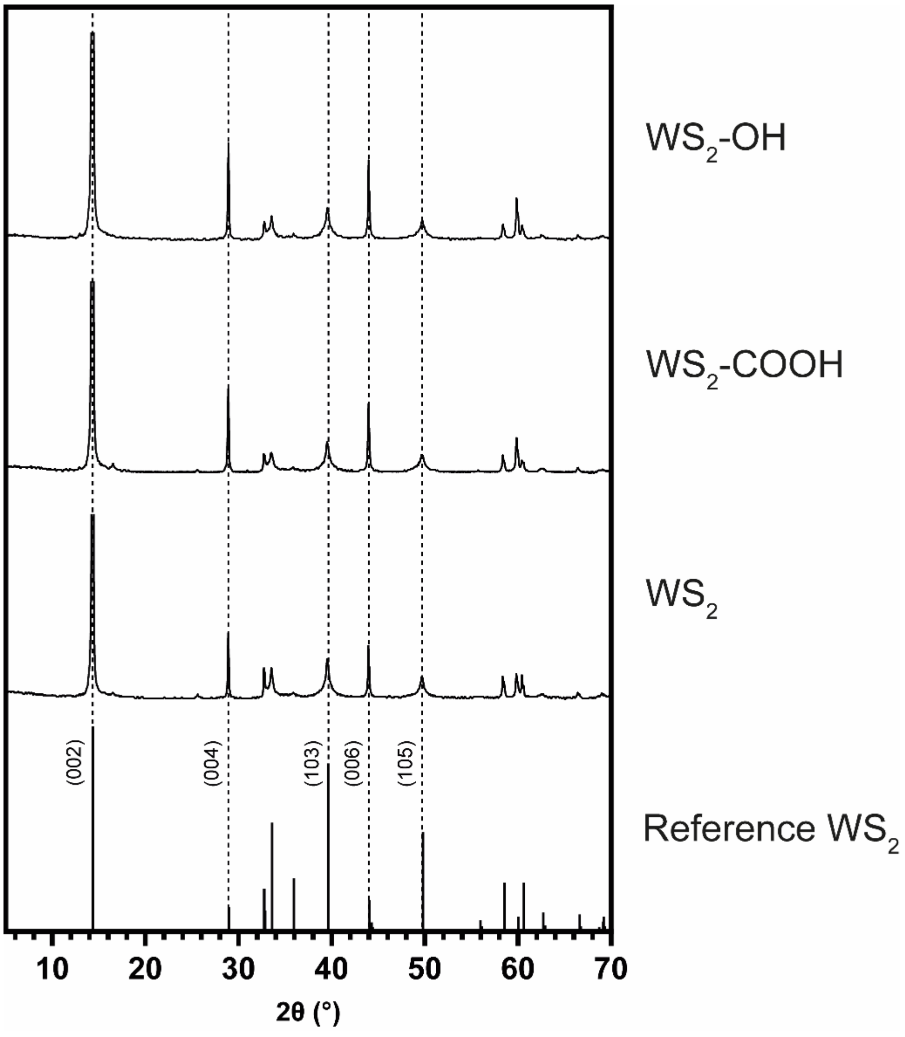

Figure S3. Experimental X-ray diffractograms of WS_2, WS_2-COOH and WS_2-OH, together with the reference WS_2 reflections and the (hkl) assignment of the main peaks used for calculating the crystallite size using the Scherrer equation.

Table S2. Position and full width at half maximum (FWHM) of the isolated peaks of XRD spectra for the different inorganic samples, as well as the corresponding crystallite size calculated using the Scherrer equation.

hkl	2θ (°)	FWHM (°)	Crystallite size (nm)
**WS_2**			
002	14.32	0.112	71.3
103	39.54	0.497	17.0
105	49.69	0.585	15.0
004	28.89	0.128	64.0
006	43.95	0.175	48.9
**WS_2-COOH**			
002	14.32	0.143	55.8
103	39.53	0.549	15.4
105	49.68	0.652	13.4
004	28.89	0.147	55.8
006	43.96	0.187	45.7
**WS_2-OH**			
002	14.33	0.142	56.4
103	39.55	0.553	15.3
105	49.7	0.634	13.8
004	28.9	0.141	58.1
006	43.97	0.185	46.4

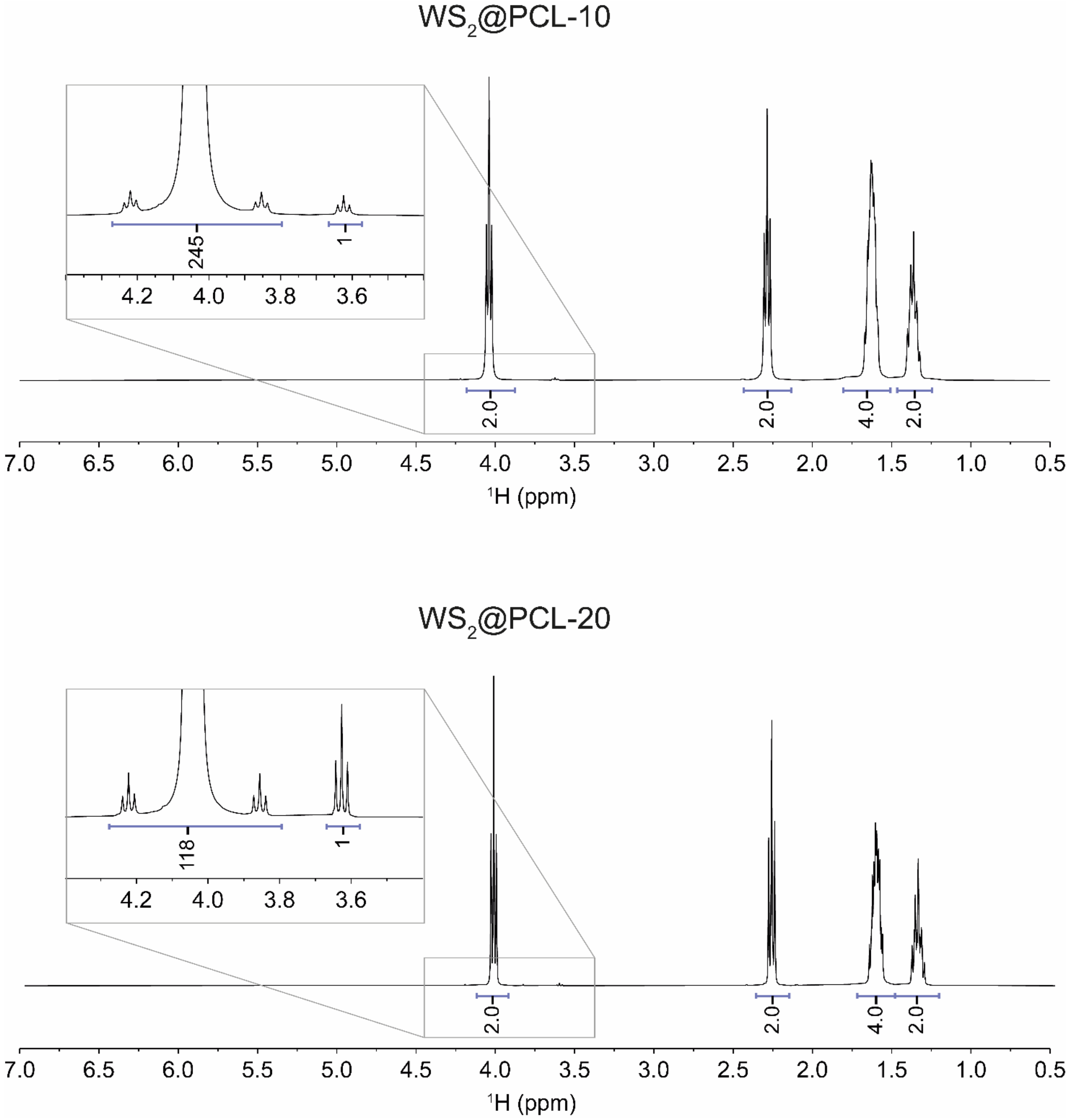

Figure S4. ^{1}H-NMR (400 MHz, $CDCl_3$) of WS_2@PCL-10 and WS_2@PCL-20, together with the expansion of the 3.4 – 4.4 ppm region with the integration of the terminal monomer peak compared to the equivalent signal of the repeating monomeric unit.

Table S3. Position and FWHM of the isolated peaks of XRD spectra for the different polymer-grafted samples, as well as the corresponding crystallite size calculated using the Scherrer equation.

hkl	2θ (°)	FWHM	Crystallite size (nm)
**WS_2@PCL-10**			
002	14.32	0.096	83.3
103	39.54	0.574	14.7
105	49.69	0.724	12.1
004	28.89	0.125	65.5
006	43.95	0.175	48.9
**WS_2@PCL-20**			
002	14.32	0.096	83.6
103	39.53	0.629	13.4
105	49.68	0.734	11.9
004	28.89	0.126	65.0
006	43.96	0.168	51.0

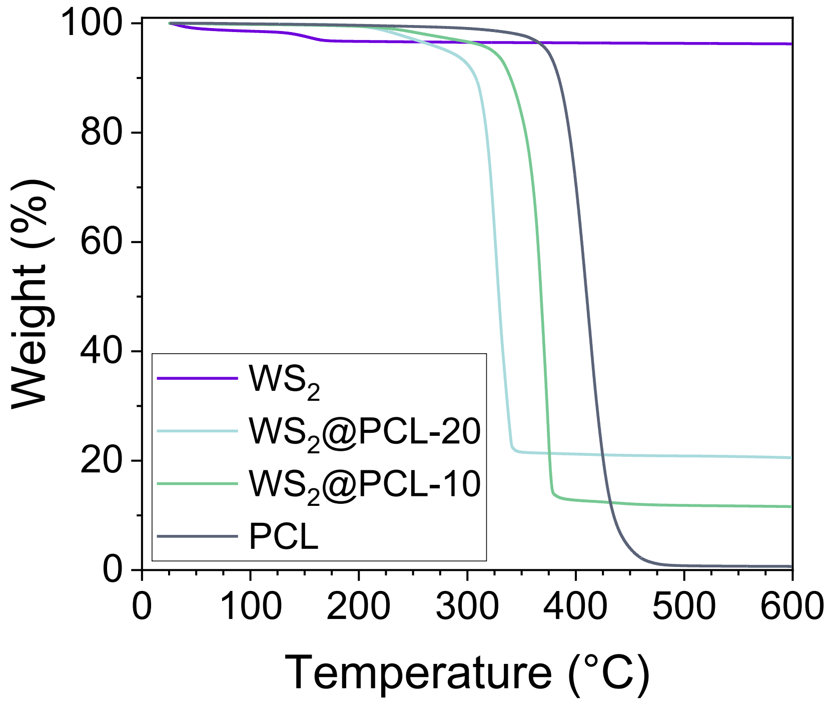

Figure S5. TGA curves of WS_2, PCL, WS_2@PCL-10 and WS_2@PCL-20.

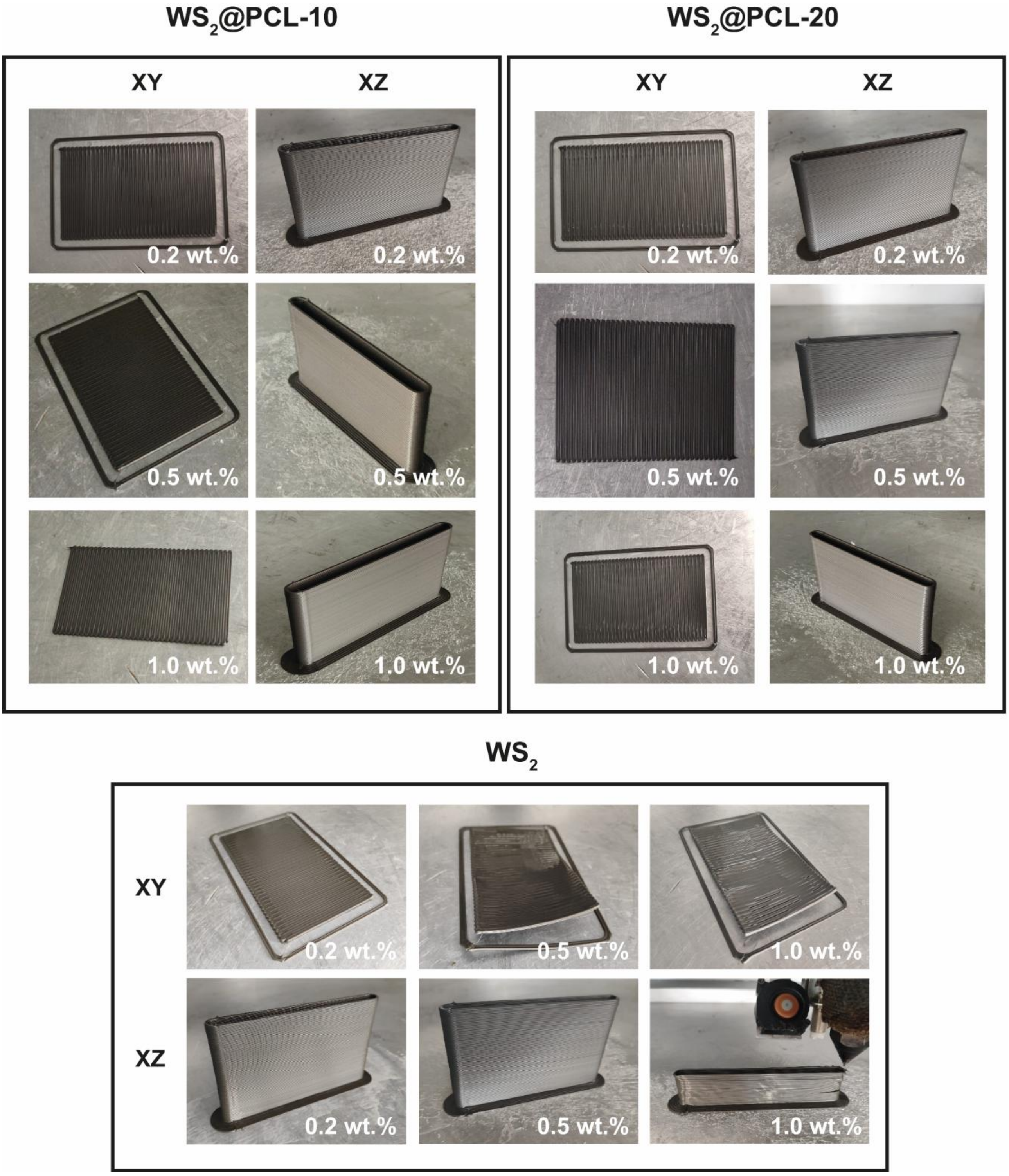

Figure S6. Photographs of FGF 3D-printed PCL nanocomposite plaques.

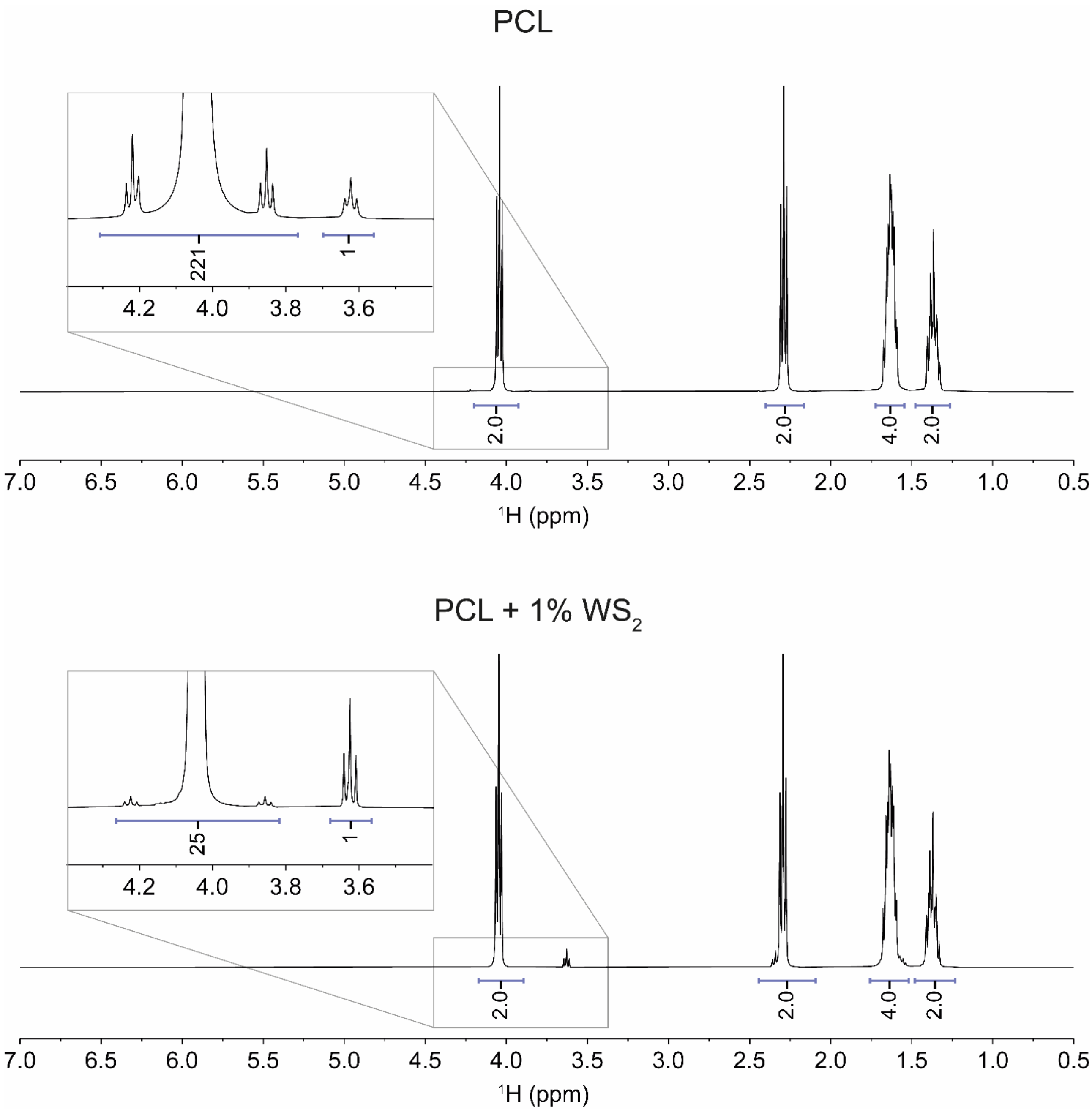

Figure S7. ^{1}H-NMR (400 MHz, $CDCl_3$) of pristine PCL and PCL + 1 wt.% unmodified WS_2, together with the expansion of the 3.4 – 4.4 ppm region with the integration of the terminal monomer peak compared to the equivalent signal of the repeating monomeric unit.

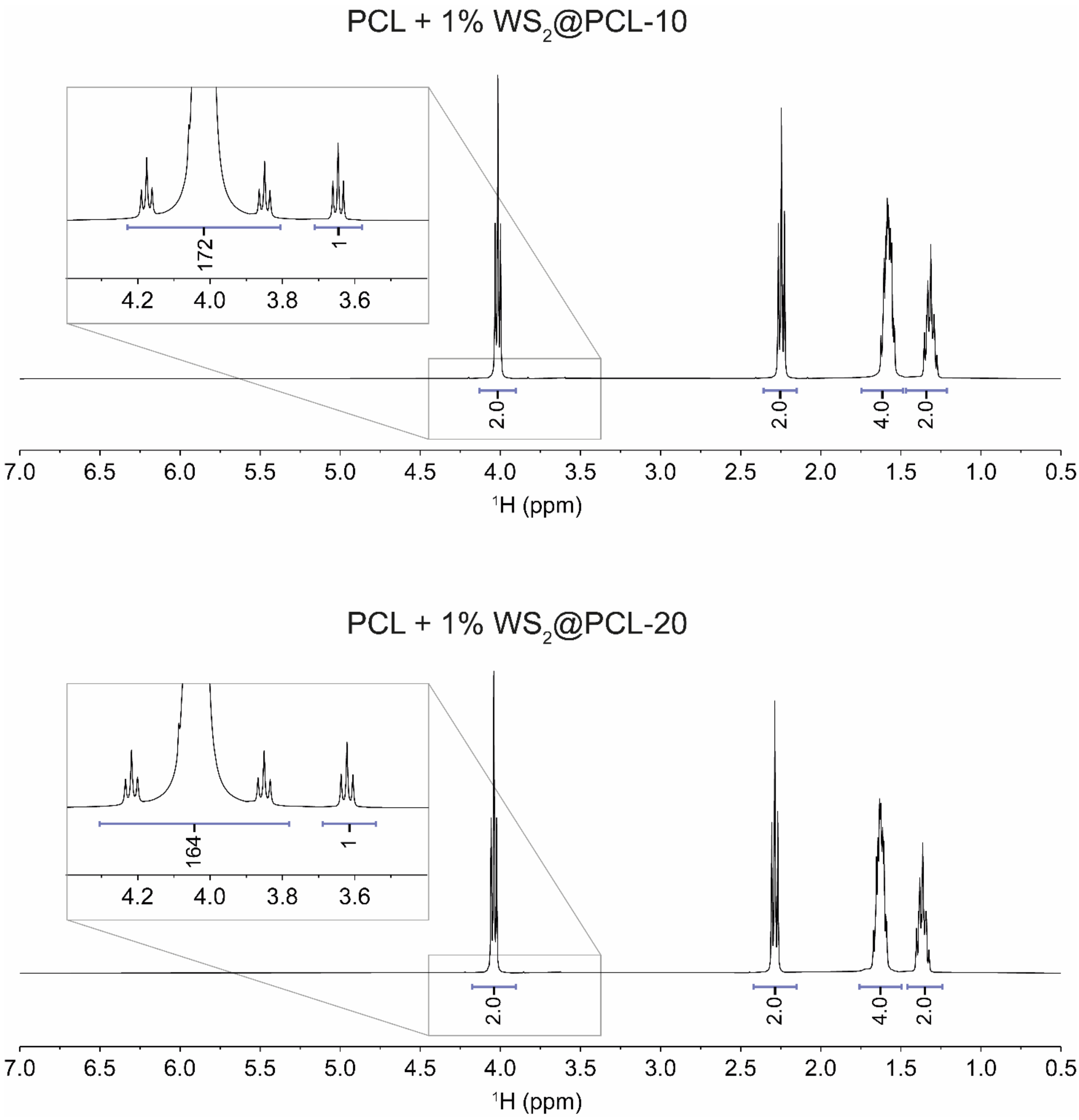

Figure S8. ^{1}H-NMR (400 MHz, $CDCl_3$) of PCL + 1 wt.% WS_2@PCL-10 and PCL + 1 wt.% WS_2@PCL-20, together with the expansion of the 3.4 – 4.4 ppm region with the integration of the terminal monomer peak compared to the equivalent signal of the repeating monomeric unit.

Table S4. Composition of the prepared WS_2 nanocomposites, as well as their measured MFR and the molecular weight (M_n) of PCL measured by ^{1}H-NMR after compounding. M_n values after 1 year of storage are also reported for the composites with the highest nanofiller loadings.

	MFR (g / 10 min)	M_n (kDa)	M_n after 1 year (kDa)
PCL	4.53 ± 0.15	50.6 ± 5.1	52.7 ± 5.3
PCL + 0.2% WS_2@PCL-10	5.14 ± 0.07	48.8 ± 4.9	-
PCL + 0.5% WS_2@PCL-10	6.63 ± 0.24	44.0 ± 4.4	-
PCL + 1.0% WS_2@PCL-10	9.57 ± 0.61	39.4 ± 3.9	38.5 ± 3.9
PCL + 0.2% WS_2@PCL-20	4.57 ± 0.22	46.8 ± 4.7	-
PCL + 0.5% WS_2@PCL-20	6.10 ± 0.15	44.8 ± 4.4	-
PCL + 1.0% WS_2@PCL-20	8.57 ± 0.34	37.6 ± 3.8	38.3 ± 3.8
PCL + 0.2% WS_2	23.7 ± 1.1	10.0 ± 1.0	-
PCL + 0.5% WS_2	36.0 ± 1.7	7.84 ± 0.78	-
PCL + 1.0% WS_2	49.5 ± 3.6	5.93 ± 0.59	6.84 ± 0.68

Table S5. Tensile properties of 3D printed nanocomposites.

XY					
**Sample**	**Young's Modulus (MPa)**	**Yield stress (MPa)**	**Yield strain (%)**	**Stress at break (MPa)**	**Strain at break (%)**
**PCL**	245 ± 8	16.3 ± 0.2	13.5 ± 0.5	25.7 ± 0.3	726 ± 14
**PCL + 0.2% WS_2@PCL-10**	291 ± 8	19.8 ± 1.0	16.1 ± 0.1	30.9 ± 1.9	682 ± 10
**PCL + 0.5% WS_2@PCL-10**	313 ± 9	20.5 ± 0.8	14.5 ± 0.5	32.3 ± 1.5	697 ± 10
**PCL + 1.0% WS_2@PCL-10**	341 ± 7	29.4 ± 0.9	14.3 ± 0.7	41.7 ± 1.2	653 ± 30
**PCL + 0.2% WS_2@PCL-20**	263 ± 6	18.6 ± 0.7	16.7 ± 1.4	30.4 ± 0.9	663 ± 8
**PCL + 0.5% WS_2@PCL-20**	291 ± 8	18.7 ± 0.6	14.9 ± 0.4	30.5 ± 1.0	705 ± 20
**PCL + 1.0% WS_2@PCL-20**	313 ± 8	20.4 ± 0.7	15.1 ± 0.2	28.8 ± 1.5	663 ± 33
**PCL + 0.2% WS_2**	278 ± 10	19.1 ± 0.7	14.1 ± 0.6	16.1 ± 1.1	351 ± 37
**PCL + 0.5% WS_2**	266 ± 49	-	-	17.1 ± 3.1	5.2 ± 1.0
**PCL + 1.0% WS_2**	-	-	-	-	-
**XZ**					
**Sample**	**Young's Modulus (MPa)**	**Yield stress (MPa)**	**Yield strain (%)**	**Stress at break (MPa)**	**Strain at break (%)**
**PCL**	277 ± 13	-	-	12.4 ± 0.3	8.1 ± 0.7
**PCL + 0.2% WS_2@PCL-10**	348 ± 11	-	-	14.6 ± 0.7	7.6 ± 0.4
**PCL + 0.5% WS_2@PCL-10**	392 ± 8	-	-	15.5 ± 0.6	7.2 ± 0.4
**PCL + 1.0% WS_2@PCL-10**	402 ± 12	-	-	20.4 ± 1.1	8.3 ± 1.0
**PCL + 0.2% WS_2@PCL-20**	328 ± 18	-	-	13.8 ± 0.5	9.0 ± 0.2
**PCL + 0.5% WS_2@PCL-20**	358 ± 10	-	-	14.6 ± 0.7	6.7 ± 0.5
**PCL + 1.0% WS_2@PCL-20**	384 ± 6	-	-	15.0 ± 0.3	7.2 ± 0.2
**PCL + 0.2% WS_2**	426 ± 33	15.1 ± 1.6	5.6 ± 0.5	5.3 ± 3.0	41 ± 18
**PCL + 0.5% WS_2**	415 ± 15	-	-	7.6 ± 0.5	1.8 ± 0.2
**PCL + 1.0% WS_2**	-	-	-	-	-

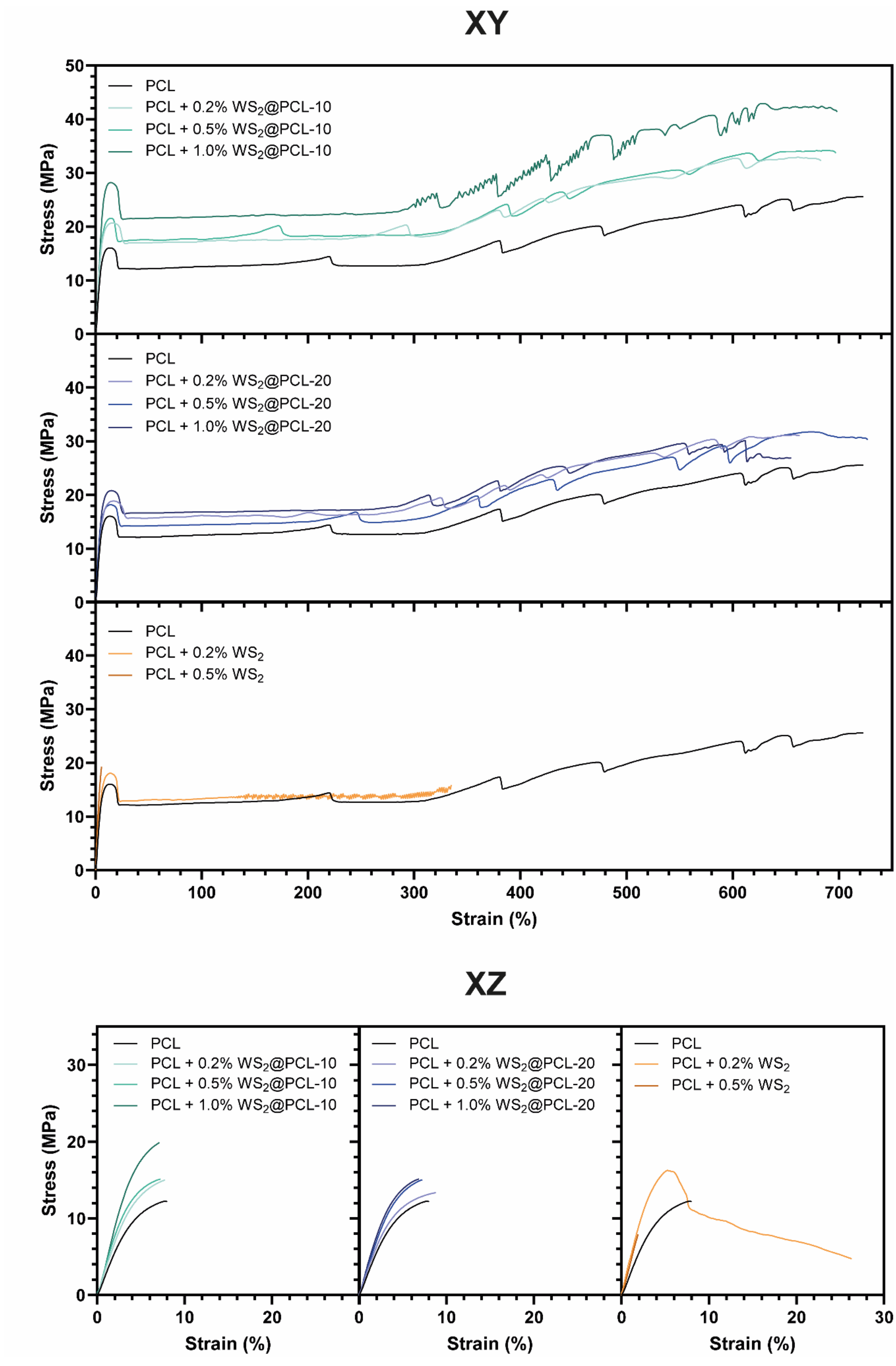

Figure S9. Representative tensile stress-strain curves.

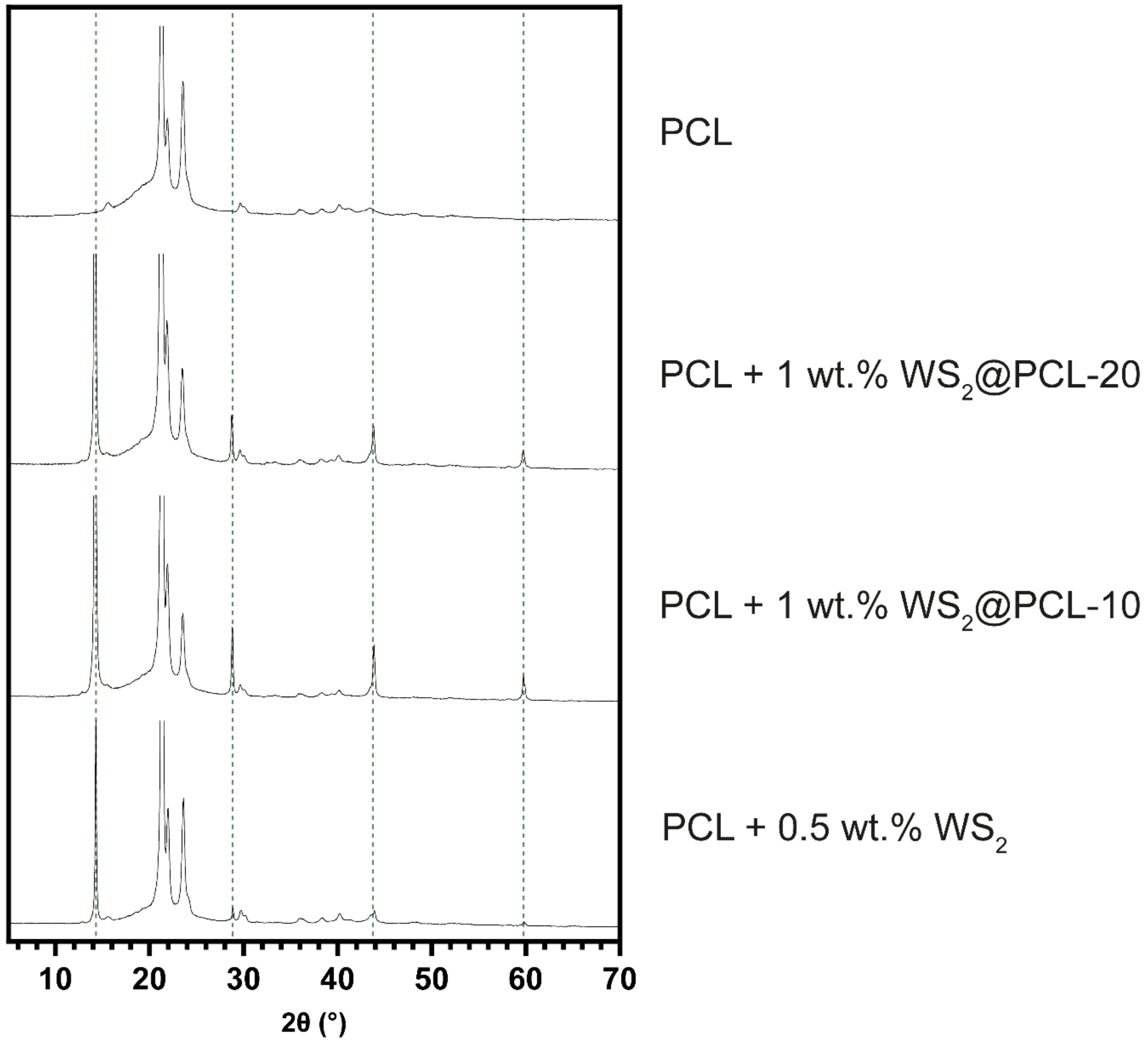

Figure S10. Experimental X-ray diffractograms of different 3D printed nanocomposites. Dashed lines correspond to the (00l) reflections of WS_2.

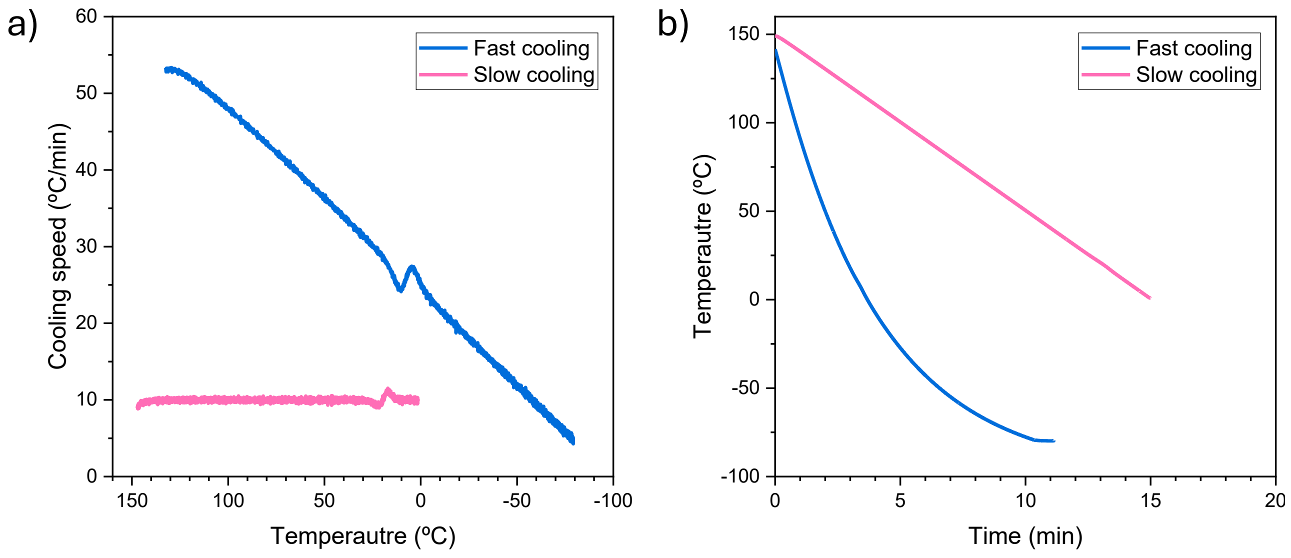

Figure S11. Temperature profiles of the two cooling steps performed by DSC. In a) is presented the dependency of the cooling speed with temperature, while b) shows the decrease of the temperature of the sample with time.

Table S6. Cold crystallization temperatures (T_{cc}) and enthalpies (ΔH_{cc}) and melting temperature (T_m) and enthalpies (ΔH_m) obtained from the DSC experiments.

	Slow cooling sweep		Fast cooling sweep		Heating sweep	
	T_{cc} (ºC)	ΔH_{cc} (J/g)	T_{cc} (ºC)	ΔH_{cc} (J/g)	T_m (ºC)	ΔH_m (J/g)
PCL	19.7	51.2	7.3	46.2	55.6	50.5
PCL + 1.0% WS_2	39.9	65.2	33.0	79.5	52.4	52.4
PCL + 1.0% WS_2@PCL-20	35.1	35.1	24.5	52.1	56.0	51.8
PCL + 1.0% WS_2@PCL-10	35.3	35.3	24.8	54.4	55.7	50.9
